# Quantitative Modeling of a Gene's Expression from Its Intergenic Sequence

**DOI:** 10.1371/journal.pcbi.1003467

**Published:** 2014-03-06

**Authors:** Md. Abul Hassan Samee, Saurabh Sinha

**Affiliations:** 1 Department of Computer Science, University of Illinois at Urbana-Champaign, Urbana, Illinois, United States of America; 2 Institute for Genomic Biology, University of Illinois at Urbana-Champaign, Urbana, Illinois, United States of America; Weizmann Institute of Science, Israel

## Abstract

Modeling a gene's expression from its intergenic locus and trans-regulatory context is a fundamental goal in computational biology. Owing to the distributed nature of cis-regulatory information and the poorly understood mechanisms that integrate such information, gene locus modeling is a more challenging task than modeling individual enhancers. Here we report the first quantitative model of a gene's expression pattern as a function of its locus. We model the expression readout of a locus in two tiers: 1) combinatorial regulation by transcription factors bound to each enhancer is predicted by a thermodynamics-based model and 2) independent contributions from multiple enhancers are linearly combined to fit the gene expression pattern. The model does not require any prior knowledge about enhancers contributing toward a gene's expression. We demonstrate that the model captures the complex multi-domain expression patterns of anterior-posterior patterning genes in the early Drosophila embryo. Altogether, we model the expression patterns of 27 genes; these include several gap genes, pair-rule genes, and anterior, posterior, trunk, and terminal genes. We find that the model-selected enhancers for each gene overlap strongly with its experimentally characterized enhancers. Our findings also suggest the presence of sequence-segments in the locus that would contribute ectopic expression patterns and hence were “shut down” by the model. We applied our model to identify the transcription factors responsible for forming the stripe boundaries of the studied genes. The resulting network of regulatory interactions exhibits a high level of agreement with known regulatory influences on the target genes. Finally, we analyzed whether and why our assumption of enhancer independence was necessary for the genes we studied. We found a deterioration of expression when binding sites in one enhancer were allowed to influence the readout of another enhancer. Thus, interference between enhancer activities was a possible factor necessitating enhancer independence in our model.

## Author Summary

Quantitative modeling of gene expression from DNA sequences and regulatory inputs underpin our studies of gene regulation. The existing literature focuses on modeling parts of a gene's expression pattern from its experimentally characterized “enhancers”, which are ∼1 Kbp long sequences, often located in the gene's intergenic region or “locus.” However, a far-reaching goal is to model all aspects of a gene's expression based on the genome sequence, without prior knowledge of enhancers. With this motivation, we have developed a model that maps a gene's locus and cellular context to its expression in that context. Using this model, we study the regulation of 27 genes having complex expression patterns in the *Drosophila* embryo. Our findings suggest the presence of sequence segments that can irreconcilably distort the gene's expression pattern and thus have to be “shut-down” by the model. We also apply the model to identify the transcription factors forming the stripe boundaries of the studied genes; and our results agree remarkably with experimental findings of decades of work. Finally, we develop a new method to analyze if and why multiple segments influencing a gene's expression need to avoid interaction between themselves.

## Introduction

Gene regulation is key to understanding of a variety of biological processes ranging from development [1] to disease [2]. Transcriptional regulation is one of the best studied stages of gene regulation [3], especially in the context of developmental biology [4]. Studies of early embryonic development in *Drosophila* [5] have revealed the roles of various transcription factors (TFs) in setting up precise spatio-temporal gene expression patterns, and delineated many “enhancers” (also called “cis-regulatory modules” or “CRMs”) that mediate the activities of combinations of TFs. We have today a fairly detailed knowledge of the transcriptional regulatory network involved in patterning of the anterior-posterior (A/P) and dorso-ventral (D/V) axes in the blastoderm-stage *Drosophila* embryo [6]–[8]. This knowledge has spurred the development of quantitative models of gene regulation that aim to map the sequence of a given enhancer to the expression pattern driven by that enhancer [9]–[17]. These models attempt to (1) predict the strength of TF binding to sites within the enhancer by using data on TF concentration and binding specificity, and (2) integrate the predicted binding strengths of multiple TFs into a quantitative prediction of that enhancer's contribution to gene expression. The prediction may vary from one cell type to another, as TF concentrations vary. The ultimate goal is to build a computational tool that automatically predicts the expression of any gene in any cellular condition based solely on the genome sequence and a quantitative description of the trans-regulatory context [18]. Such a computational tool will embody our knowledge of the so-called “cis-regulatory code” [19], [20]. It will help us annotate the regulatory genome at a single nucleotide resolution, and predict the effects of genotypic changes (in cis or in trans) on gene expression and phenotype.

### Gene expression modeling

In this study, we consider the problem of *modeling* gene expression, which is an important intermediate step in the more ambitious goal of building the *predictive* tool mentioned above. In the modeling task, we are given the inputs (sequence and TF concentrations) and output (gene expression), and a model with tunable parameters is trained to map the inputs to the outputs. Such a model has many possible uses. Once trained on wild-type data on a gene, it can be used to predict outputs on non-wild-type inputs, which may include changes in cis (sequence) or trans (TF concentrations). It can provide a quantitative description of how a specific gene's regulation is encoded in the sequence, and can precisely characterize each TF's role in regulating the gene. Moreover, by testing alternative models in terms of their goodness-of-fit on the data, we can gain valuable insights into the mechanisms underlying gene regulation. Here, we develop such a model of gene expression, show that it fits the available data accurately, and demonstrate a few practical utilities of the model.

### Locus-level gene expression modeling

**Figure 1:**
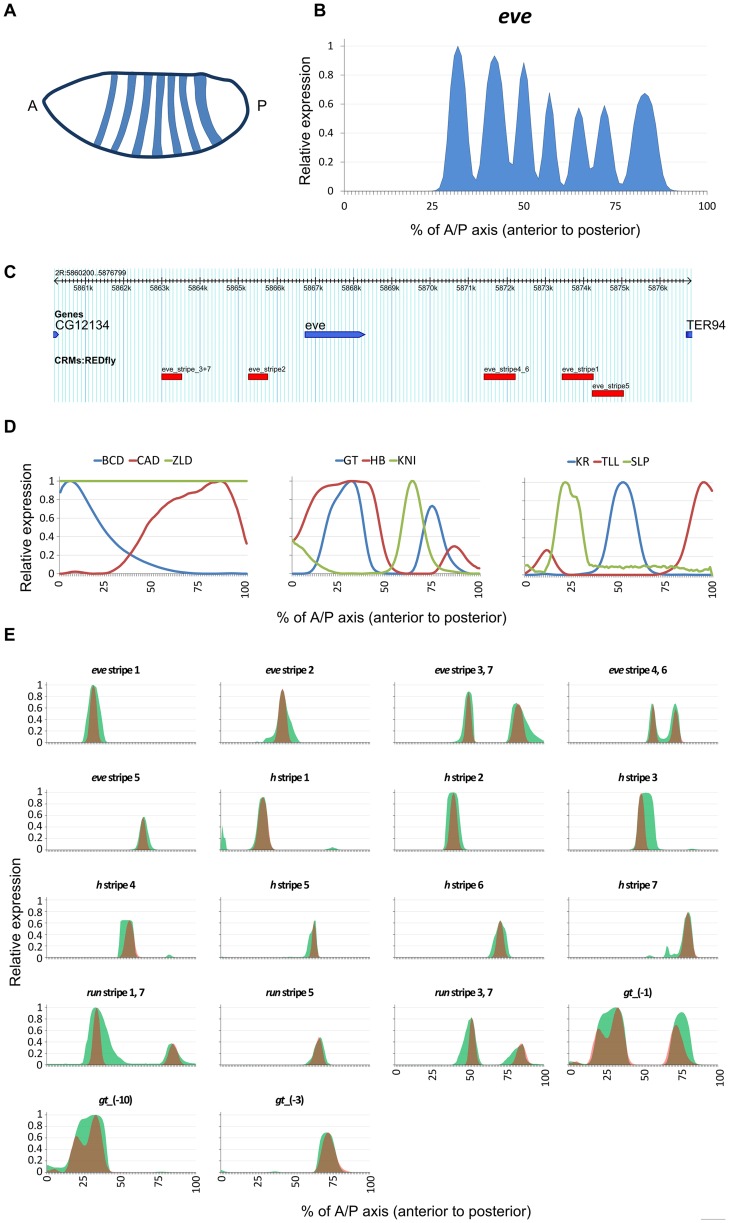
(A) Schematic of expression pattern of the pair-rule gene *even-skipped* (*eve*) in *D. melanogaster* embryo. ‘A’ and ‘P’ denote the anterior and the posterior ends of the embryo, respectively. (B) Quantitative profile of *eve* gene expression along the anterior-posterior axis of the embryo. (C) Genome Browser view of the five distinct enhancer elements that drive *eve* gene expression; each enhancer's name denotes the specific stripe(s) of gene expression that it drives. The entire locus is 17 Kbp long. (D) Concentration profiles along the anterior-posterior axis, for the nine TFs used to model the expression patterns of the genes *eve*, *h*, *run*, and *gt*. (E) Real (red) and GEMSTAT-predicted (green) expression profiles along the A/P axis for the known enhancers of *eve*, *h*, *run*, and *gt*.

A key challenge in achieving the above-stated goal is to model a gene's expression *from the sequence of its entire intergenic region*, or “locus”. While regulatory influences on a gene have been known to be located at great distances (>1 Mbp) from the gene [21], [22], it is frequently observed that much of the information about the gene's expression pattern is encoded in its locus [23]. This information is typically organized in modular units of length ∼1 Kbp, called enhancers, that are scattered in the locus, both proximal and distal to the gene, and upstream, downstream as well as within introns of the gene. For instance, complex gene expression patterns such as the seven-striped patterns of “pair-rule” genes (Figure 1A,B) in the *Drosophila* embryo are known to be determined by multiple, distinct enhancers (Figure 1C), each of which is sufficient to drive a discrete aspect (one or two stripes) of the gene's overall pattern [24], [25]. How the information encoded by multiple enhancers in a locus is integrated together is a largely unexplored problem. A simple hypothesis might be that the binding sites located across different enhancers in a gene's locus constitute one large enhancer, interpreted by the same rules of combinatorial action that apply to binding sites within any single enhancer. The more common view [26], [27], however, is that each enhancer is interpreted independently of others, and readouts of multiple enhancers are superimposed or combined additively to produce the gene expression pattern. If this latter view is more accurate, existing sequence-to-expression models, which have been tested on individual enhancers, may not suffice to model a gene's expression from its entire intergenic region. Indeed, while there have been several successful attempts to model enhancer readouts, especially for A/P and D/V patterning genes in *Drosophila* [9]–[18], [28], we are not aware of any computational model that has been successfully tested on a multi-enhancer gene locus such as those of the pair-rule genes (Figure 1A–C). Our primary objective in this work is to implement and test such a computational model. A recent study by Kim et al. [18] makes significant contributions to this modeling question, although the authors' primary focus was on elucidating specific details of transcriptional control mechanisms. (Also see Discussion.)

We present a computational framework for modeling the expression level of a gene from the sequence of its locus and a quantitative description of the trans-regulatory context (TF concentrations). We refer to this task as “locus-level gene expression modeling”, where a gene's locus is considered to be the non-coding sequences extending upstream and downstream of the gene until a neighboring gene's boundary. (This includes UTRs and introns.) Our new model, called GEMSTAT-GL (“**G**ene **E**xpression **M**odeling based on **Sta**tistical **T**hermodynamics - **G**ene-locus **L**evel”), implements the two-layered, modular organization of cis-regulatory information mentioned above, thus reflecting the commonly held view today.

### Practical problems in implementing a locus-level model

An important challenge for a model that interprets multiple enhancers in a locus and combines their separate readouts is the unknown location of enhancers in a locus [29]–[31] – an enhancer is typically ∼1 Kbp long and may be located anywhere within the much longer (often 10–50 Kbp long) gene locus. Accurate identification of all the necessary enhancers in the locus will be a prerequisite for modeling gene expression. High throughput characterization of chromatin marks [32]–[35] and computational enhancer scans [29], [36]–[40] may help overcome this challenge in the future; but ideally the quantitative model should automatically discover the contributing segments in the locus, rather than relying on enhancers identified *a priori*. A second major challenge in locus-level modeling is to model the mechanisms that integrate outputs from distinct enhancers into the endogenous gene expression. As noted above, a relatively simple “additive” mechanism has been suggested in the literature [26], [41]–[43], where readouts of the contributing enhancers are summed up to produce the gene expression pattern. However, existing quantitative models often are capable of predicting enhancer readouts only on a relative scale (e.g., expression pattern along the A/P axis rather than absolute expression values). As such, it is not clear if a simple summation of model predictions on enhancers will suffice to accurately predict gene expression patterns. Moreover, while a minimal set of enhancers may capture all aspects of the gene expression pattern, it is not clear what role the rest of the locus plays. If the locus harbors multiple enhancers with similar readouts, as has been suggested by the discovery of “shadow enhancers” [44]–[46], a quantitative model should take into account contributions from all of them. These are some of the challenges related to locus-level gene expression modeling that motivate our work.

### Overview of model development and testing

**Figure 2:**
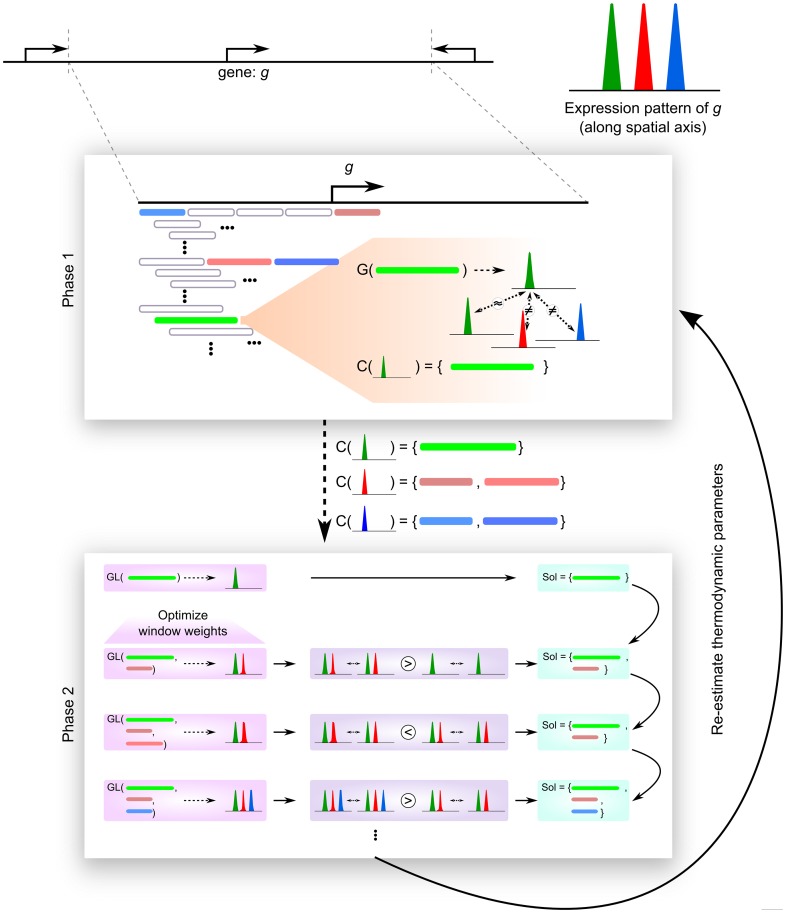
Overview of the newly proposed GEMSTAT-GL model (details are given in Materials and Methods). The model has two types of parameters, namely thermodynamic parameters (to compute the expression readout of any sequence window) and window-weight parameters (to compute a weighted summation of the expression readouts of a set of selected windows). The parameters are optimized iteratively to fit the expression pattern of a given gene from its locus. In the example shown, GEMSTAT-GL is applied to fit the three-striped expression pattern (shown by the green, the red, and the blue stripes) of a gene *g* from its locus. Each iteration in model training consists of two phases. In the first phase, through a sliding window mechanism, the model selects a set *C(s)* of candidate windows for each stripe *s*. To this end, each window's readout (computed by GEMSTAT, denoted here by function *G*) is compared against each individual stripe, as exemplified through the operations on the green window. (Computation of the initial estimates for thermodynamic parameters is explained in main text.) In the second phase, a solution is constructed by iteratively checking if including a new window from the candidate sets (computed in Phase 1) improves model performance. In the shown example, the green window first gets included in the solution since it fits the green stripe satisfactorily. Next, the first window from the red stripe's candidate set is added to the solution and weights for the two windows are optimized so that a weighted summation of their readouts (denoted by function *GL*) fits the expression pattern consisting of the green and the red stripes. The model shows improved performance and hence, the red window is retained in the solution. However, when the second window from the red stripe's candidate set is added to the solution, it deteriorates model performance. The window is therefore discarded. Similarly, the blue window from the blue stripe's candidate set is checked and found to improve model performance – resulting in its inclusion to the solution. After completing the second phase, the model re-estimates the thermodynamic parameters and loops back to Phase 1.

We report here the first general-purpose quantitative model of a gene's expression pattern as a function of the sequence of its entire locus. Here, “general-purpose” implies that the model can be trained on any given data set with minimal or no manual parameter tuning. Admittedly, the model has to be provided with a complete set of candidate regulators (TFs), as well as their DNA binding motifs and relative concentrations, which currently limits its applicability to regulatory networks where such information is available. But given this information the model then automatically learns values for all of its free parameters, and the locations of relevant enhancers in the gene locus. As noted above, the new model treats the expression readout of an entire gene locus as being two tiered – 1) sites within each enhancer act together to produce that enhancer's contribution, which is modeled using the thermodynamics-based GEMSTAT model of enhancer function [11], and 2) contributions from multiple enhancers are combined as a weighted sum to produce the gene expression profile. See Figure 2 for an overview of the newly proposed GEMSTAT-GL model. We initially focused on the expression patterns of the genes *even-skipped*, *hairy*, *runt*, and *giant* in the developmental stage following the maternal to zygotic transition [6] in early *Drosophila melanogaster* embryos. In this stage, each of these genes is expressed in a complex multi stripe pattern and is known to be regulated by multiple enhancers within its locus, and is thus an ideal test case for locus-level modeling. As a point of contrast to the two-tiered model of GEMSTAT-GL, we also trained the GEMSTAT model that was shown previously to accurately model ∼40 enhancers involved in A/P patterning. We found that GEMSTAT fails to model multiple sharply defined stripes and instead predicts one broad expression domain when it is used for locus-level modeling of each of the four genes mentioned above. In order to demonstrate the broader applicability of GEMSTAT-GL, we next used it to model the expression patterns of 23 additional genes in early *Drosophila* embryo (we have thus modeled all the 27 A/P genes from Kazemian et al. [13]). From the intergenic locus of each gene, our model automatically selected one or a handful of segments that together generated the gene's expression. The selected segments were found to overlap enhancers known to regulate the gene, even though the model was not informed about these enhancers. We also investigated whether and how the intergenic sequence outside these selected segments contributes to the gene's expression. Our findings suggest the presence of sequence segments in the locus that would exert an irreconcilable impact on the gene's expression pattern and thus were required to be explicitly “shut down” by the model, presumably reflecting a similar phenomenon *in vivo*.

### Practical utilities of the new model

We used our models to analyze several aspects of the regulation of *eve*, *h*, *run*, and *gt*. 1) An immediate practical benefit of our model is the automatic discovery of candidate enhancers in the locus, along with accurate assignments of regulatory activity to each enhancer. This goes one step beyond our previous work [13] where enhancers were annotated based on their pattern generating potential. The new method ensures that activities of multiple enhancers in the locus can be aggregated to match the gene's expression profile. Also, since GEMSTAT-GL allows model parameters to be trained simultaneously with the discovery of enhancers in a gene's locus, the assignment of regulatory activity to enhancers is empirically more accurate than those reported in [13]. 2) We performed *in silico* knock-downs of TFs and identified the TFs responsible for the formation of stripe boundaries in A/P expression patterns of these genes. The resulting network of regulatory interactions exhibits a very high level of agreement with known regulatory influences on the target genes, illustrating the potential of the model-based approach for unraveling regulatory networks. 3) We also developed a method to investigate whether and why the assumed independence of enhancers was necessary in our model. We found that interaction or “cross-talk” [43], [47]–[49] between the enhancers of a gene is detrimental to our model's fits to the gene's expression data, and identified cases where specific binding sites in one enhancer that may interfere with another enhancer's readout. This suggests that in these cases the independence of enhancer contributions is necessary for proper modeling of gene expression.

An implementation of the “GEMSTAT-GL” model is available for download at: http://veda.cs.uiuc.edu/gemstat-gl/index.htm.

## Results

### A thermodynamics-based model accurately predicts readouts of the enhancers of *even-skipped*, *hairy*, *runt*, and *giant*

We previously reported a statistical thermodynamics-based model of enhancer function, called “GEMSTAT”, that was shown to successfully predict the expression patterns of ∼40 enhancers of the anterior posterior (A/P) axis patterning system in early *Drosophila* embryo [11]. GEMSTAT is built on basic physical principles laid out by Shea and Ackers [10], [50]. It is the only available general purpose tool that can predict the expression readout of an arbitrary DNA segment and whose parameters can be trained on any given set of enhancers. It assumes gene expression in a cell type to be proportional to the fractional occupancy [51] of the basal transcriptional machinery at the gene promoter, and estimates this occupancy from the enhancer sequence and the binding specificities (motifs) and concentrations of TFs in that cell type. Due to its previous successful application to individual enhancers and due to our extensive experience with it, GEMSTAT was a natural initial choice for modeling a gene locus. We made a major modification to GEMSTAT's objective function, which is used to compare predicted and real expression patterns: instead of the “root mean square error” function [11], it now uses a “weighted Pattern Generating Potential” (w-PGP) function [52] that was designed specifically for comparing spatial gene expression patterns. (See Materials and Methods and Figure S10 for details.)

**Figure 3:**
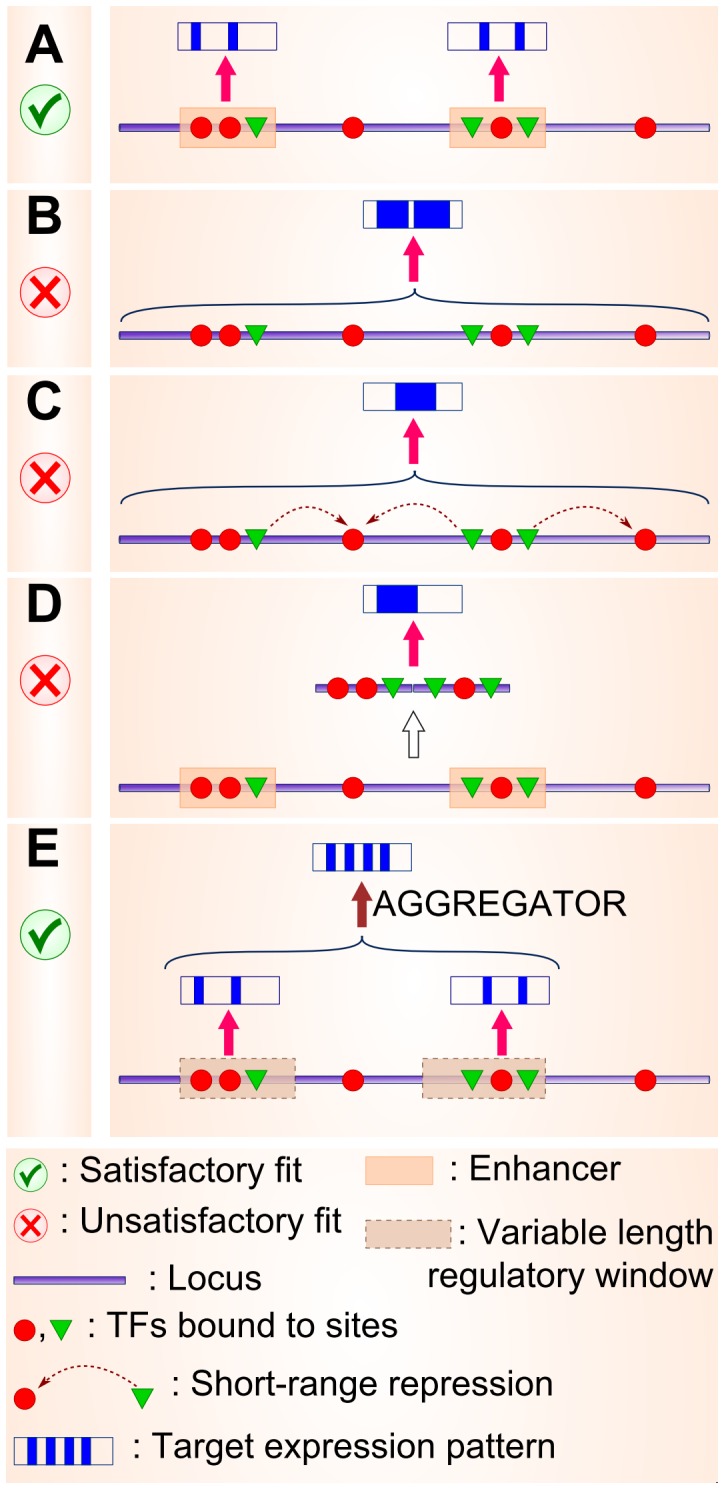
A hypothetical example illustrating the different attempts at developing a locus-level model of gene expression. Notations used in the figure are explained in the bottom panel. The hypothetical gene here is expressed in four stripes, as shown in the panel for notations using four blue stripes within a rectangle. The thick purple line near the base of each panel denotes the locus; red circles and green triangles denote activator and repressor TFs bound to their cognate sites within the locus, respectively. The bold pink arrow indicates GEMSTAT prediction of an expression readout on a given segment. (A) GEMSTAT accurately models the 2-striped expression patterns driven by “known” enhancers for this hypothetical gene. (B) GEMSTAT fails to model the four-striped readout of entire locus in the “Direct Interaction” mode. (C) GEMSTAT fails to model the locus readout in the “Short Range Repression” mode (quenching effect of repressors is shown using the dark-red colored dashed arrows connecting repressors to activators bound at nearby sites). (D) GEMSTAT also fails to model the gene's four-striped expression from the concatenation of its two “known” enhancers. (E) A two-tiered model, that first selects a handful of variable-length windows (putative enhancers) from the locus and then takes a weighted summation of the GEMSTAT-predicted readouts of those windows to model gene expression. This model produces accurate fits.

Before using GEMSTAT to model the entire locus, we sought to confirm if it accurately models the characterized enhancers of the genes of interest in this study. We first focused on four genes in the early *Drosophila* embryo, namely *even-skipped (eve)*, *hairy (h)*, *runt (run)*, and *giant (gt)*. The multi-stripe patterns of these genes (e.g., Figure 1A,B) are among the first manifestations of complex combinatorial regulation in the *Drosophila* embryo [53]. These genes are initially regulated by an interplay of maternally deposited proteins and their immediate regulatory targets [6], and their expression is later stabilized through more complex mechanisms including auto-[54], [55] and cross-regulation [56]. Due to the complexity and multi-enhancer origins of their expression patterns and due to availability of high resolution expression data [57], these four genes were chosen as the primary subject of our study. (See Text S1 for a discussion of why several other complex patterned genes were not included in the initial study.) A total of 18 early functioning enhancers have been reported in the literature for *eve*, *h*, *run*, and *gt* – 5 for *eve* (Figure 1C), 7 for *h*, 3 for *run*, and 3 for *gt* – each responsible for some discrete aspect (typically one or two “stripes”) of the respective gene's pattern during early stages of development. Thus, for each gene our data set included the sequences and known expression readouts of each enhancer, and the DNA motifs and A/P concentration profiles of nine TFs – BCD, CAD, ZLD, GT, HB, KNI, KR, TLL, and SLP (Figure 1D) – that are known to regulate expression at this stage of development [6], [58], [59]. For each gene, GEMSTAT learns one set of parameters so as to maximize the agreement between predicted and known expression profiles of all enhancers of the gene according to the w-PGP metric (see Materials and Methods). As shown in Figure 1E, readouts of known enhancers were modeled accurately for each of the four genes, suggesting that the GEMSTAT model captures the combinatorial action of multiple, heterotypic binding sites in those enhancers. (The enhancers responsible for stripes 2, 4, and 6 of *run* are not known.) This exercise is shown schematically in Figure 3A. We used a constrained parameter estimation strategy here to guard against over-fitting. (See Materials and Methods.)

### Intergenic locus readout under the thermodynamic model does not agree with multi-stripe expression pattern

Having confirmed that GEMSTAT can model enhancer readouts accurately, we next tested if GEMSTAT can model the multi-stripe patterns of the genes of interest from their respective intergenic regions (Figure 3B). By doing so, we hoped to answer the following question raised in the introductory section: Do the rules for interpreting a collection of binding sites in an enhancer apply unchanged to the larger collection of sites present throughout the locus? The intergenic region or “locus” was defined here as the sequence bounded by the immediate neighboring genes on either side (Figure 1C), and was of length 17 Kbp, 68 Kbp, 58 Kbp, and 17 Kbp for *eve*, *h*, *run*, and *gt*, respectively (Table S1).

We performed two exercises, under different assumptions about the range of regulatory influence of repressors. In the first exercise, we assumed that repressor molecules bound to their cognate binding sites can directly affect the transcriptional machinery (“DIRECT INTERACTION” mode of GEMSTAT [11]). However, as shown in Figure S1A, GEMSTAT was unable to find any set of parameters for which the predicted gene expression profiles match the multi-stripe profiles. One possible explanation for this failure is the phenomenon of “short range repression” (SRR). Some of the repressors of this regulatory system (e.g., GT, KNI, and KR) are known to act over short ranges only, i.e., their binding sites mediate a repressive action only if located within 100–150 bp of activator sites [28]. Therefore, in our second set of tests we trained GEMSTAT in the “SRR” mode [11], which captures short range repression, on each gene's locus (Figure 3C). However, this test was also unsuccessful (Figure S1B), i.e., no parameter setting was found for which predicted expression profiles match the real gene expression profiles. We note that all of these failed experiments were performed with an unconstrained parameter estimation strategy (which is GEMSTAT's default strategy, see Materials and Methods). Therefore, failures of these experiments were presumably not due to shortcomings of the parameter optimization algorithm.

The finding that GEMSTAT successfully models enhancer functions but fails on the entire locus has at least two possible explanations. The first explanation is that binding sites within certain segments in the locus contribute to gene expression while sites outside of these segments do not contribute, and their inclusion in the model is somehow detrimental to the goodness-of-fit. To test this, we concatenated the known enhancers of each gene (Figure 3D) and searched for the best fit between GEMSTAT predictions and data. No satisfactory fit was found (Figure S1C), suggesting that the above explanation is not sufficient. A second explanation for the failure of GEMSTAT on locus-level modeling has to do with the way GEMSTAT models the sequence. It computes the readout as a single non-linear function of (the strengths of) all binding sites in the sequence. Perhaps the readout of the locus is not best described as computing this function on all sites in the locus, even though the readout of individual enhancers does conform to this model. An emerging hypothesis was that local clusters of sites act together in ways captured by the GEMSTAT model (as demonstrated by the enhancer modeling exercise above) but contributions from different clusters of sites do not interfere with each other and these clusters should not be interpreted together. This hypothesis reflects the conventional wisdom about cis-regulatory architecture, and was reached here on the basis of the failed modeling exercises described above. We explored this hypothesis next, within a modeling framework, and found it being supported by all the genes modeled in this work.

### A two-tiered model based on GEMSTAT accurately predicts expression from the entire gene locus

Our working hypothesis now was that distinct segments in the gene locus are interpreted separately based on the collection of sites within each segment, and their individual readouts are then aggregated to produce the overall pattern. Thus, it presents a “two-tiered” gene expression model. The main challenges in formulating and training such a model are: (i) determining the segments whose readouts are aggregated, and (ii) choosing an appropriate aggregator function. The quantitative model may not assume prior knowledge of enhancers in the locus since such a strategy is not generalizable to poorly characterized loci. Gene expression profiles should be modeled solely from the gene locus and TF data (concentrations and motifs).

Pursuing the above hypothesis, we implemented a two-tiered model that uses contributions from a number of sequence windows in the locus, and predicts gene expression as a weighted sum of these contributions (Figure 3E; see Figure 2 for more details). We call this new model “GEMSTAT-GL”, with “GL” abbreviating for “gene-locus level”. The sequence windows were allowed to be of varying lengths, even mutually overlapping if necessary, and their separate readouts were predicted using GEMSTAT. The number and locations of contributing sequence windows, as well as the weight of each window's contribution were left to be automatically discovered during model training. Model training was performed iteratively, with a new sequence window being included for contributing to gene expression only if its inclusion significantly improved the agreement between predicted and real expression profiles. In this way, the complexity of the model was kept under control. Details of this two tiered model and its parameter estimation procedure are described in Materials and Methods. Roughly speaking, this procedure (a) finds a window whose GEMSTAT readout matches one aspect (e.g., a stripe) of the gene expression pattern, (b) tests if a weighted summation of this window's readout and the readouts of already selected windows improves the overall prediction, and (c) includes the window if such an improvement is noted. The model parameters were fit separately for each gene; hence we adopted a “constrained” parameter estimation strategy to avoid over-fitting (see Materials and Methods and Discussion).

**Figure 4:**
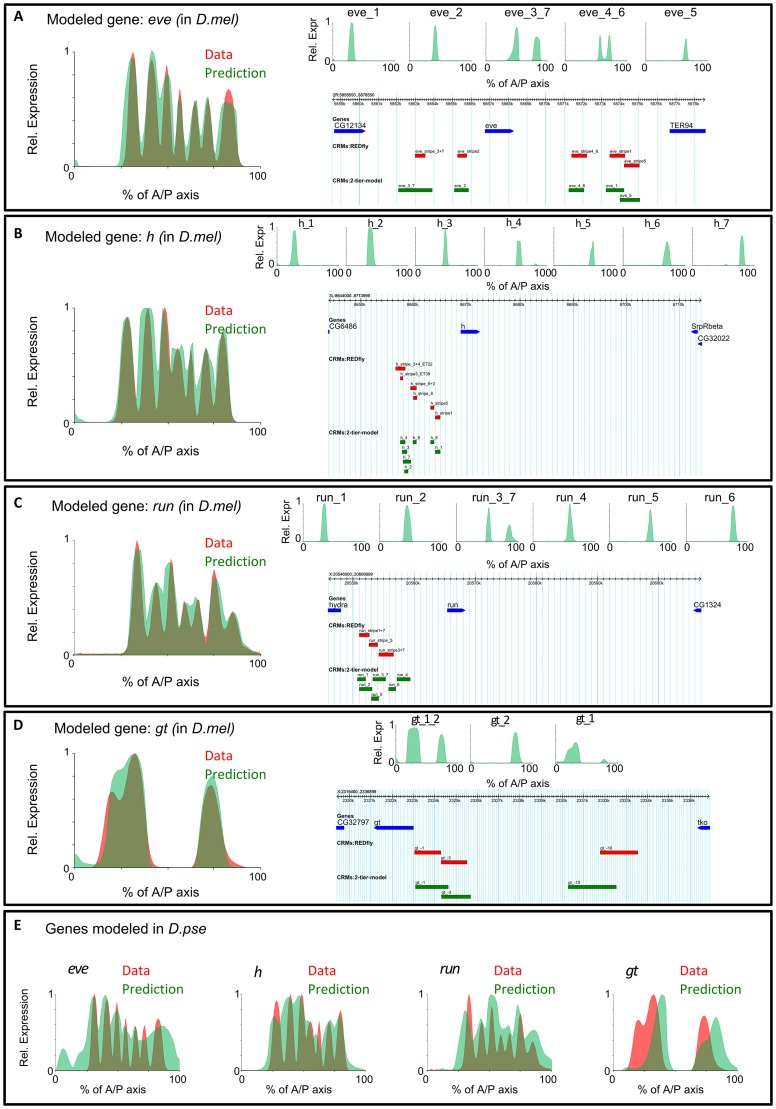
(A) Results of applying GEMSTAT-GL on the intergenic region of *eve* in *D. melanogaster.* Left panel shows the real (red) and predicted (green) expression profiles along the A/P axis. Right panel shows the locations of selected windows (green boxes) in the locus and their predicted expression patterns (top), along with locations of known *eve* enhancers (red boxes). (B), (C), and (D), same information for *h*, *run*, and *gt*, respectively. (E) Expression patterns modeled by GEMSTAT-GL from the intergenic regions of *eve*, *h*, *run*, and *gt* in the *D. pseudoobscura* genome.

Predictions from the GEMSTAT-GL model agreed very well with the real expression profiles of each of the four target genes, *eve*, *h*, *run*, and *gt* (Figure 4A–D). For instance, we noted that the seven-stripes of *eve* and *h* expression were faithfully captured by the model (Figure 4A,B), while the seven-striped pattern of *run* was well approximated by a six-striped predicted pattern, with the model failing to separate stripes 4 and 5. Both domains of *gt* expression and their experimentally characterized assignments to three different enhancers were reproduced by the model. The agreement between model and data seen here for the *eve* and *h* stripes is qualitatively superior to corresponding fits in previous work on enhancer modeling [11], [14], and this may be attributed to the fact that GEMSTAT-GL fits parameters on each gene separately. However, subsequent control experiments (described next) largely ruled out the possibility of obtaining such accurate models through over-fitting and highlighted the significance of the reported models. From each gene's locus, the model chose a small number of segments (at most seven) in the first tier before aggregating their GEMSTAT-based readouts in the second tier. The segments selected from a locus received comparable weights, with their values differing by at most two-fold (see Figure S2). Moreover, these automatically chosen segments showed strong overlap with previously characterized enhancers of the respective genes (Figure 4A–D and Figure S3), even though the enhancers were not known to the model training procedure. In particular, of the 21 regulatory segments chosen from the four gene loci, 16 overlapped with REDFly enhancers [60]. The extent of overlap between GEMSTAT-GL selected regulatory segments and REDFly enhancers are shown in Figure S3.

**Figure 5:**
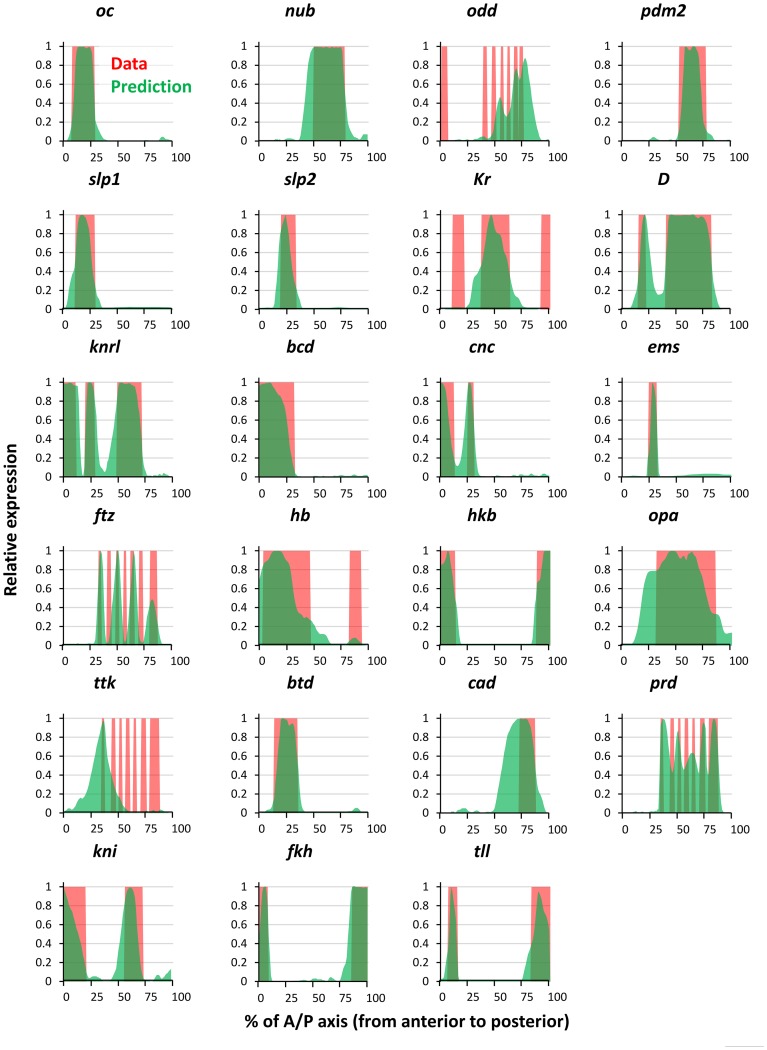
Results of fitting the GEMSTAT-GL model on the intergenic locus of 23 additional genes studied in [13]. Quantitative data on target expression patterns were obtained from the companion website of the same study, and were originally derived from *in situ* expression images at the FlyExpress [81] database. For each gene, the red and the green plots represent the target (real) and the modeled expression patterns, respectively.

This initial success of the model motivated us further to test its generalizability. We therefore applied the model to all 27 A/P genes considered in [13]. These 27 genes, which are expressed between stages 4 and 6 during *Drosophila* embryogenesis, include several gap genes, pair-rule genes, and anterior, posterior, trunk, and terminal genes. They are, with the exception of secondary pair-rule genes (Text S1), likely to be regulated primarily by the maternal and the early zygotic proteins, and therefore are reasonable targets for modeling using the same input TFs as above. (We also used the TFs Capicua (CIC), Forkhead (FKH), and Huckebein (HKB) in modeling these genes, as in [13].) The four genes modeled above – *eve*, *h*, *run*, and *gt* – are included in these 27 genes; hence we show the modeled expression patterns of the additional 23 genes in Figure 5. GEMSTAT-GL was able to accurately fit the expression pattern for most of the genes, demonstrating its wide applicability for gene-locus modeling. The model fits were less accurate for the secondary pair-rule genes *ftz*, *odd*, and *prd*, where 4, 3 and 5 stripes were correctly reproduced (out of seven stripes of each gene). This relative lack of accuracy is probably because the direct regulators of these genes include the primary pair-rule proteins [61], which were not among the input TFs (see Text S1). Another case of model failure was *ttk*, presumably because the precise seven-striped pattern of *ttk* occurs later than stage 6 of embryogenesis [62] and it requires other regulators than the used TFs (e.g., Biniou [63]). To model these additional 23 genes, GEMSTAT-GL selected 29 regulatory segments, 23 of which were overlapping with REDFly enhancers (see Figure S3 for the extent of overlap). As above, a constrained parameter fitting strategy was used here. The w-PGP scores of GEMSTAT-GL for all the 27 genes modeled above are shown in Table S2.

#### Control experiments suggest that the trained model is not over-fit

Over-fitting was a concern in the above modeling exercise, since our framework does not allow testing of predictions on unseen data. We performed a number of control experiments, described next, to address this concern. As “negative controls”, we repeated the above model-training exercise on the following types of artificial data sets (see Materials and Methods): (a) the locus of one gene was used to model the expression pattern of a different gene, (b) the locus of a given gene was used to model a “random” expression pattern, and (c) a gene's expression pattern was modeled from a randomly generated sequence of the same length as the gene's locus, and (d) a gene's expression pattern was modeled from a random relocation of TF binding sites in its locus. Each negative control experiment failed, as expected: no parameter settings were found for which model predictions agreed with data (Figure S4). Moreover, experiment (d) allowed us to assess the significance of our original model fits by comparing the goodness-of-fit score (value of objective function) of the trained model to an empirical distribution of scores from 100 negative controls for each gene. As shown in Figure S5, the original models were highly significant, with goodness-of-fit scores greater than all negative controls and with values 30–40 standard deviations above the mean from negative controls. We note that, as opposed to the constrained parameter estimation strategy in the modeling of real data, there was no constraint on parameter values in the control experiments. As an additional test, we trained the model on *D. melanogaster* gene expression profiles of *eve*, *h*, *run* and *gt* using sequence from the loci of their respective *D. pseudoobscura* orthologs. We assumed that the expression profile characterized experimentally in *D. melanogaster* remains unchanged in this related species [14]. The trained model was found to capture the real expression profiles well (Figure 4E), although not as accurately as in *D. melanogaster*: for the seven-striped patterns of *eve*, *h*, and *run*, the model reproduced the locations of 6, 7, and 6 stripes respectively, though the inter-stripe boundaries were not as prominent as in the *D. melanogaster* models. The model fits on *gt* reproduced both anterior and posterior domains of endogenous expression, though the model-predicted domains were shifted posteriorly. We note again that we are unable to test the trained model by direct prediction of the readout of an unseen gene locus, since the locations and weights of contributing sequence windows have to be learned from that locus.

### A sampling strategy reveals the cis-regulatory architecture of a gene locus

**Figure 6:**
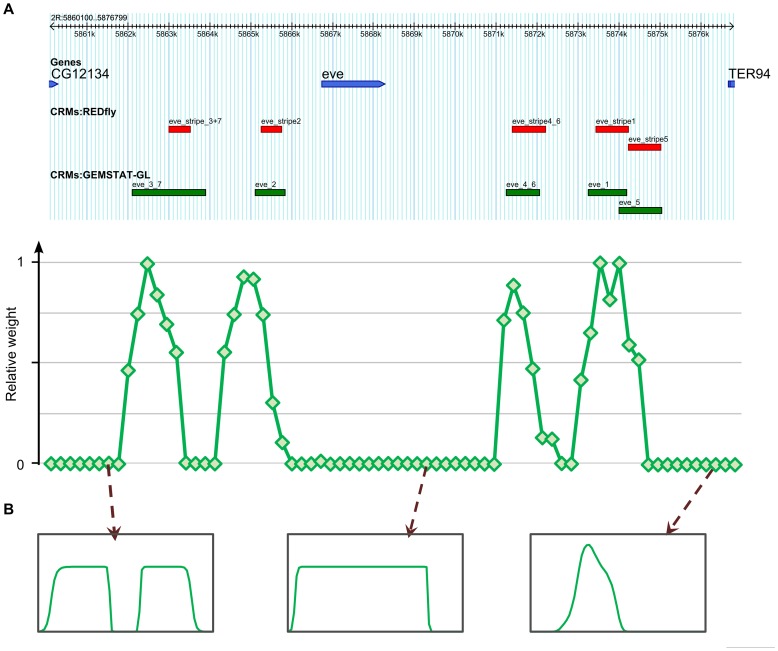
Outcome of MCMC sampling to reveal the cis-regulatory architecture of *eve* intergenic region. (A) Top panel shows the *eve* intergenic locus along with the known enhancers of *eve* and windows selected by GEMSTAT-GL to model *eve* expression pattern. Bottom panel shows the average weight of segments in the locus as estimated by MCMC sampling. The horizontal axis of the bottom panel spans the *eve* locus; green diamonds in the plot represent the starting positions of the sequence segments that comprise the MCMC samples (segments corresponding to two different green diamonds might therefore differ in length). The vertical axis denotes the average weight (on a relative scale between 0 and 1) that each segment received over 50,000 samples. (B) Predicted readouts of three zero-weight segments that could have an irreconcilable effect on the gene expression pattern, and were not selected by the two-tiered model.

The two-tiered model described above discovered a small number of segments whose readouts could be aggregated to match the gene expression profile. This set of segments describes the “regulatory architecture” of the gene locus (Figure 4A–D), as a checkered pattern of putative enhancers (green boxes in the genome browser views) interspersed with large spacer regions that do not contribute to gene expression. However, since the model was trained with a local search algorithm and was designed to utilize only as many segments as necessary, it is possible that the learned architecture is one of many possible architectures, each of which has its own locations of putative enhancers and intervening spacers. To investigate this possibility, we performed Markov Chain Monte Carlo sampling of the space of architectures. (See Materials and Methods for details.) Each architecture was represented by the locations of sequence segments that contribute to gene expression, and their respective weights. Also, each architecture was sampled with probability proportional to its w-PGP score, which quantifies how well the model predictions for that architecture agree with gene expression. A summary of the large number (50,000) of architectures sampled by this scheme from the *eve* locus is shown in Figure 6A. Similar depictions for the *h* and *run* locus are given in Figure S6. It shows the average weight that a segment received over all samples. (A weight of zero indicates that the segment was part of the spacer regions between putative enhancers in that architecture, and weights cannot be negative.) We see that the average weights are heavily peaked at a handful of locations, while most other segments within the locus have very low average weights. Moreover, the high weight locations are coincident with the contributing segments from the optimal architecture found above (Figure 4A). This indicates the existence of a unique regulatory architecture at the gene locus. We also noted that the high weight segments of this architecture overlap known enhancers of the gene.

On the other hand, there were many segments with average weight close to 0 (Figure 6A), that were not included in any sampled architecture. Such segments either (a) have no regulatory information within them, or (b) their readout as predicted by the GEMSTAT model is inconsistent with and must not be aggregated with the readouts of other segments. The latter possibility suggests that there may be segments that exert an irreconcilable impact on the gene's expression pattern and thus have to be explicitly “shut down” by the model. A direct examination of their predicted readouts confirmed that this was indeed the case for some segments (Figure 6B). While most of the non-contributing segments had no noticeable readout, some such segments led to predicted expression at levels comparable to the known enhancers but at inappropriate axial positions, i.e., outside the stripe domains.

### A regulatory network of transcription factors determining “stripes” of gene expression

One of the advantages of a quantitative model of gene expression is that it allows us to predict the effects of perturbations in *cis*- (the regulatory sequence) or in *trans*- (the transcription factors) on expression. For example, a “*knock-down*” of a TF is easily simulated by setting the TF's concentration to zero. Such *in silico* knock-downs may then be used to infer regulatory influences of any TF on the gene, and a transcriptional regulatory network may be constructed. In our past work [13], we constructed such a regulatory network at the level of individual enhancers, i.e., the network predicted when a TF's knock-down would significantly affect an enhancer's readout. Such an effect does not necessarily translate to a change in gene expression, as there may be redundancy of information in the locus [44], [46], [64]. An advantage of having a quantitative model of the readout of the entire gene locus is that regulatory networks may be constructed at the level of genes rather than enhancers. An edge in such a network would correspond to a TF's knock-down affecting the gene expression; such an effect can be then be probed experimentally through an *in situ* hybridization assay in *TF^−^* condition. (Testing a TF-enhancer association experimentally would involve reporter gene assays, which are more expensive.)

**Figure 7:**
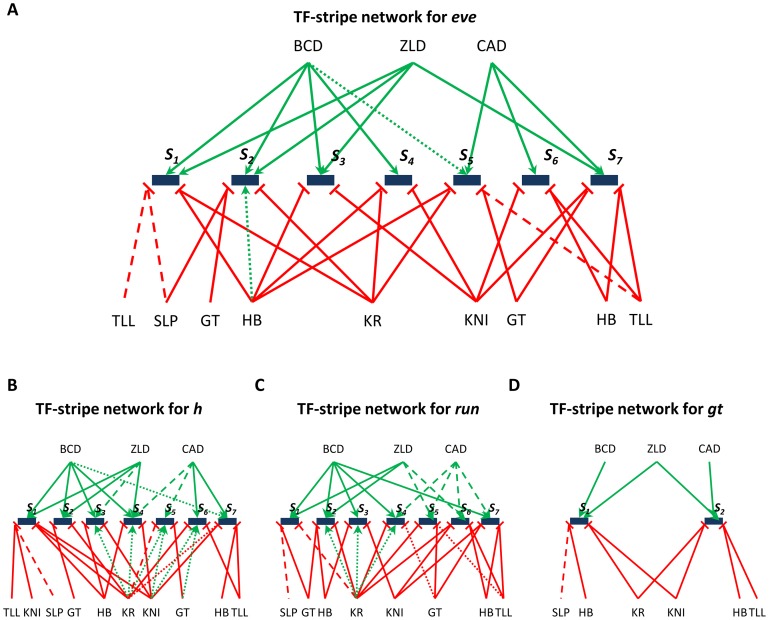
(A)–(D) Networks showing regulatory influences of TFs on individual stripes of *eve*, *h*, *run*, and *gt*, respectively. Red edges denote repressive and green edges denote activating role of the corresponding TF. Solid edges denote predicted influences that are already known in the literature. Edges with large dashes denote predicted influences that were not reported in the literature before (false positive or novel predictions), while edges with small dashes denote predicted influences already known in literature but missed by our model (false negatives).

Here, we used *in silico* knock-downs to predict TF-gene regulatory interactions, and described the predicted interactions as a “TF-stripe” network where edges connect TFs to specific stripes in the gene's expression profile, reflecting an effect of the TF on establishment of that particular stripe. The TF-stripe network for the *eve* gene (Figure 7A) shows 35 edges (12 activating, 23 repressive influences) between nine TFs and seven stripes of *eve* expression. The activators BCD and CAD regulate the anterior and posterior stripes, as expected from their concentration profiles. Each of the two borders (anterior and posterior) of any stripe is regulated by one or two TFs. This automatically constructed network is in very high agreement with the literature: 30 of the 35 edges have been previously confirmed or hypothesized based on genetic evidence, and only two interactions (small dashed edges: BCD→Stripe 5 and HB→Stripe 2) with experimental evidence were not recovered by our procedure. The “HB→Stripe 2” interaction cannot be recovered by our model because we assign a fixed role (activator or repressor) to each TF, while the literature points to an activating role for HB at stripe 2 [65], [66] and a repressive role elsewhere [67]. Overall, the strong agreement between the predicted and previously characterized TF-stripe network strongly argues for the usefulness of our approach, when we consider the vast amount of experimental work that has gone into characterizing those 30 recovered edges. Moreover, our model-based approach predicts three regulatory interactions that were not known previously (large dashed edges). These include roles for TLL and SLP in setting up the anterior border of Stripe 1 and a role for TLL at the posterior border of Stripe 5. Similar TF-stripe networks were constructed for *h*, *run*, and *gt*; these networks are shown, along with known interactions from the literature, in Figure 7B–D. As in the network for *eve*, we missed very few of the known edges in these latter three networks, and most of the missed edges correspond to “indirect” activation (i.e., if A is a repressor of B and B is a repressor of C, then A indirectly activates C) which can only be captured by a network level model of gene regulation [68].

A comparison of the networks predicted by GEMSTAT-GL with the ones deduced in previous computational studies [13], [53] highlights several edges that previous models had failed to identify but have been corroborated by *in vivo* experiments. For example, the “TLL→*h* Stripe 6” and “TLL→*h* Stripe 7” edges in our network were suggested previously through experiments involving *tll* mutant embryos [25], [69], but the enhancer-based model of our previous work [13] misses both of these edges. Several such examples were also noted with respect to the network reported in [53] (not shown).

An important observation from the TF-stripe networks of Figure 7 is the major role played by Zelda in setting up pair rule gene expression. Recent studies have shown Zelda (Zld) to be a master regulator of early embryonic development [58], [59], [70], and Nien et al. [59] have specifically shown the effect of Zelda knockdown on pair-rule expression. While all four genes (*eve*, *h*, *run*, and *gt*) showed severely modified expression in *zld^−^* experiments, a closer examination of Figure 5 in [59] reveals specific effects that are in agreement with our TF-stripe network. For instance, the *h* gene shows complete abolishment of stripes 1, 2, 4, consistent with our predictions of direct Zelda influence on stripes 1, 2, 3, and 4 of this gene. Similarly, the most pronounced effect of Zelda knockdown on *run* expression is the abolishment of stripes 1, 2, 5, and 6, and our network predicts direct effects of Zelda of stripes 1 and 2. We are not aware of any previous computational modeling effort that predicts these specific effects of Zelda.

We should note that, our reported success in recapitulating known regulatory edges is based on our own literature survey where we have tried to be as exhaustive as possible, but admittedly we might have missed some results. As such, the high rate of recapitulated network edges is a preliminary, rather than an absolute, assessment of the accuracy of these networks.

### Modeling cross-talk between enhancers results in aberrant expression readouts

Several studies make a case for interactions between enhancers of a gene [47]–[49], [71]–[74], raising doubts about enhancer modularity or independence [44], [75]. Our experience in computational modeling of gene expression, as reported above, seems to suggest that enhancer independence is the common case. GEMSTAT-GL, which assumes independence of enhancer activities and linear aggregation of their readouts, fits expression data accurately, while GEMSTAT, which interprets all binding sites in the locus together, completely failed to fit the data. We investigated the source of this dichotomy in a systematic way, by modifying GEMSTAT-GL to allow for a limited degree of interaction (non-independence) between enhancers and noting cases where such interaction leads to a marked deterioration in model fits. We report this analysis for the enhancers of *eve*, *h*, *run*, and *gt*.

Let C_1_ and C_2_ be two non-overlapping enhancers (and the only two enhancers) in a locus. Let *E* denote the gene expression profile and let G(C_i_) denote the readout predicted by GEMSTAT for any enhancer C_i_. As described in the previous sections, GEMSTAT-GL tests how well C_1_ and C_2_ explain *E* by computing w-PGP(*E*, G(C_1_)+G(C_2_)), i.e., the similarity between gene expression profile E and the integrated output of C_1_ and C_2_. (We ignore weights of summands here, for simplicity.) Now, let us consider any sub-segment *c* of C_2_ and represent by G(C_1_, *c*) the GEMSTAT prediction on the set of binding sites in C_1_ and *c* considered together. This simulates an interaction between C_1_ and a part of C_2_. We may now use w-PGP(*E*, G(C_2_)+G(C_1_,*c*)) as the accuracy of a model where the outputs of C_1_ and C_2_ are no longer independent, and in particular, the output of C_1_ is shaped by contributions from a part of C_2_. Let G_1_ and G_2_ denote two GEMSTAT-GL models (i.e., two different parameter settings) trained to optimize w-PGP(*E*, G_1_(C_2_)+G_1_(C_1_)) and w-PGP(*E*, G_2_(C_2_)+G_2_(C_1_,*c*)), respectively. Our goal is to find a *c* such that w-PGP(*E*, G_2_(C_2_)+G_2_(C_1_,*c*))<w-PGP(*E*, G_1_(C_2_)+G_1_(C_1_)), i.e., where the model with enhancer interaction is significantly worse than the additive model. Likewise, we search for a subsegment *c* of C_1_ such that w-PGP(*E*, G_3_(C_1_)+G_3_(C_2_,*c*))<w-PGP(*E*, G_1_(C_1_)+G_1_(C_2_)) where G_3_ is a new GEMSTAT-GL model trained to optimize w-PGP(*E*, G_3_(C_1_)+G_3_(C_2_,*c*)). The discovery of any such subsegment of either C_1_ or C_2_ will point to an *avoided interaction* between the two enhancers, i.e., a specific example in support of the enhancer independence assumed in GEMSTAT-GL.

**Figure 8:**
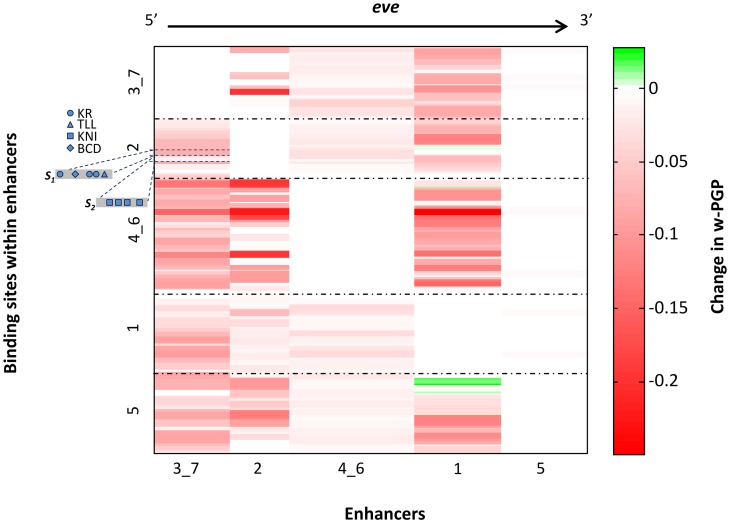
A heatmap visualization of the changes in GEMSTAT-GL's goodness-of-fit owing to interactions between the enhancers selected for the *eve* gene. The heatmap has 5 columns and 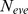 rows, where 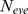 denotes the total number of binding sites in the five *eve* enhancers. Each row in the heatmap represents a binding site; the ordering of the rows, from top to bottom, reflects the 5′ to 3′ order of the respective binding sites in the locus. Each horizontal dot-dash line demarcates binding sites from two different enhancers. Each column in the heatmap represents an enhancer; the columns are ordered, from left to right, according to the 5′ to 3′ order of the corresponding enhancers in the locus. The cell at row *i* and column *j* represents, on a green-to-red color scale (green: high, red: low), the effect of allowing the binding site *i* to interact with enhancer *j*. This effect quantifies how the goodness-of-fit improves (green) or decreases (red) when interactions are allowed (see Materials and Methods for details). Two segments *S_1_* and *S_2_* within the *eve* stripe 2 enhancer are shown on the left of the heatmap, along with their constituent binding sites for TFs KR, TLL, KNI, BCD. Each of these segments has binding sites that, when allowed to interact with the eve_3_7 enhancer, result in poorer fits.

We show in Figure 8, using a heat map, the outcome of the above analysis performed on the five enhancers contributing towards the *eve* gene's expression. Rows in this heat map represent binding sites within the enhancers, and columns represent enhancers. The cell at row *i* and column *j* represents the effect (on model fits) of allowing the binding site *i* to interact with enhancer *j*. Red indicates that modeling this interaction leads to worse fits, suggesting that the interaction is avoided in reality through unknown mechanisms of enhancer independence. Green color in the heat map suggests a synergistic interaction.

Heat maps for the four genes modeled in this study (Figure 8 and Figure S7) highlighted the necessity of their enhancers to act autonomously. The many red cells indicate that such interaction must be explicitly avoided. For instance, we noted that a segment containing KR sites within the *eve* stripe 2 enhancer (Segment *S_1_*, Figure 8) adversely affects the predicted readout of the *eve* stripe 3+7 enhancer. These KR sites, when included in modeling the stripe 3+7 enhancer result in a weaker stripe 3, since the expression domain of KR covers *eve* stripe 3. A similar effect is noted for a second segment in the stripe 2 enhancer (Segment *S_2_*, Figure 8) that contains four KNI sites, which adversely influence modeling of the stripe 3+7 enhancer. Although the latter contains several KNI sites, the four additional KNI sites impart more repression than necessary and hence a deterioration in the quality of fit. (This deterioration is, however, less severe than that caused by the first segment.) These examples provide more detailed insights into why we failed in our initial attempts to model gene expression from an entire locus using GEMSTAT, where all such interactions were allowed.

## Discussion

We have presented for the first time a quantitative model that relates gene expression to the sequence of an entire gene locus, using information on the trans-regulatory context (TF concentrations). We started by showing that the thermodynamics-based model “GEMSTAT” accurately models individual enhancer readouts, but fails to model the entire locus. We then performed a series of tests where we changed the way the GEMSTAT model was applied to the locus, all of which resulted in failure. We developed a new model called GEMSTAT-GL where the expression readout of the locus is two-tiered: sites within each enhancer act together to produce that enhancer's contribution, and contributions from multiple enhancers are aggregated to produce the gene's expression pattern. This model shows very good fits to the data (Figure 4 and Figure 5) for the 27 genes studied here, and most remarkably for the complex, seven-stripe patterns of *eve*, *h*, and *run*. The process of training the model on a gene locus automatically predicts enhancers in that locus, without relying on chromatin accessibility data, and makes accurate assignments of regulatory activity to each of the predicted enhancers. We will make available, upon publication, a general-purpose implementation of the GEMSTAT-GL model that may be applied to any gene for which the relevant inputs (TFs, TF motifs, TF concentrations) and output (gene expression) are known. The implementation also allows users to include chromatin accessibility data as a filter on the locus being modeled.

We note that the GEMSTAT-GL model, as presented here, is given an intergenic sequence and its expression readout, and it finds a plausible explanation of whether and how the sequence could drive that expression. As such, it can be applied, in principle, to any of the thousands of genes whose embryonic expression patterns are known from *in situ* hybridization assays [76]. Once trained, the model reveals the cis-regulatory architecture of the locus (locations and readouts of individual enhancers), and can predict the effects of perturbations in cis (sequence) or trans (TF concentration).

However, the model cannot currently be used to predict the expression readout of a gene locus from sequence only. This is because the locations of contributing segments in the locus are free parameters of the model and can be learnt only if the gene expression readout is known. Thus, the model performance reported here refers only to “training data accuracy”, and leaves open the possibility of over-fitting. However, the model training failed on a variety of different “negative control” tests, where there was no link between the given sequence and expression, thus addressing concerns of over-fitting. We expect future work to address the current limitation that prevents the new model from a full-fledged application to the genome. One way this may be achieved is through intelligent use of accessibility and chromatin state information [33], [77] from the locus when selecting segments that contribute to gene expression.

Another potential limitation of this work is its reliance on prior knowledge of the TFs relevant to the regulatory system being studied (the A/P patterning system here). Ideally, the model should be able to automatically identify the TFs that are needed to explain the data, but this ability was not tested in this work. In a separate work [52], we address the question of systematically identifying the TFs to use when modeling enhancers using GEMSTAT.

A basic principle underlying GEMSTAT-GL is the modular view of the gene locus' readout, which holds that individual enhancers drive discrete aspects (e.g., one or two stripes) of the gene's expression pattern, through combinatorial action of the binding sites within them, and the overall gene expression pattern results from a superposition of these separate enhancer readouts. Our tests showed that a model that violates this modular view and instead interprets all binding sites in the locus as acting together is unlikely to fit the data. In other words, the rules for interpreting the set of sites across all enhancers are not the same as the rules that apply to sites within an enhancer. The final subsection of RESULTS provides details of this principle in action: the different enhancers have the potential to interfere with each other, i.e., if some sites in one enhancer, say S_i_, are interpreted together with sites of another enhancer, say S_j_, the combined readout may be different from the readout of S_j_ itself.

Another defining aspect of our model is the use of a “weighted sum” as the aggregator of multiple enhancer readouts. We note that the weights assigned by the model to different contributing segments (enhancers) are comparable to each other (Figure S2), and that a simple unweighted sum captures the seven stripe pattern of gene expression qualitatively (Figure S2), but fails to capture the “valley” between stripes 2 and 3 for *eve* and between stripes 4 and 5 for *run*; whereas the prediction for *h* remains relatively unaffected. Thus, the use of non-uniform weights may be a way for our model to correct for inaccuracies of the GEMSTAT model in predicting enhancer readout, especially at stripe borders. These weights need not be a reflection of any fundamental biochemical preference for one enhancer over another.

**Figure 9:**
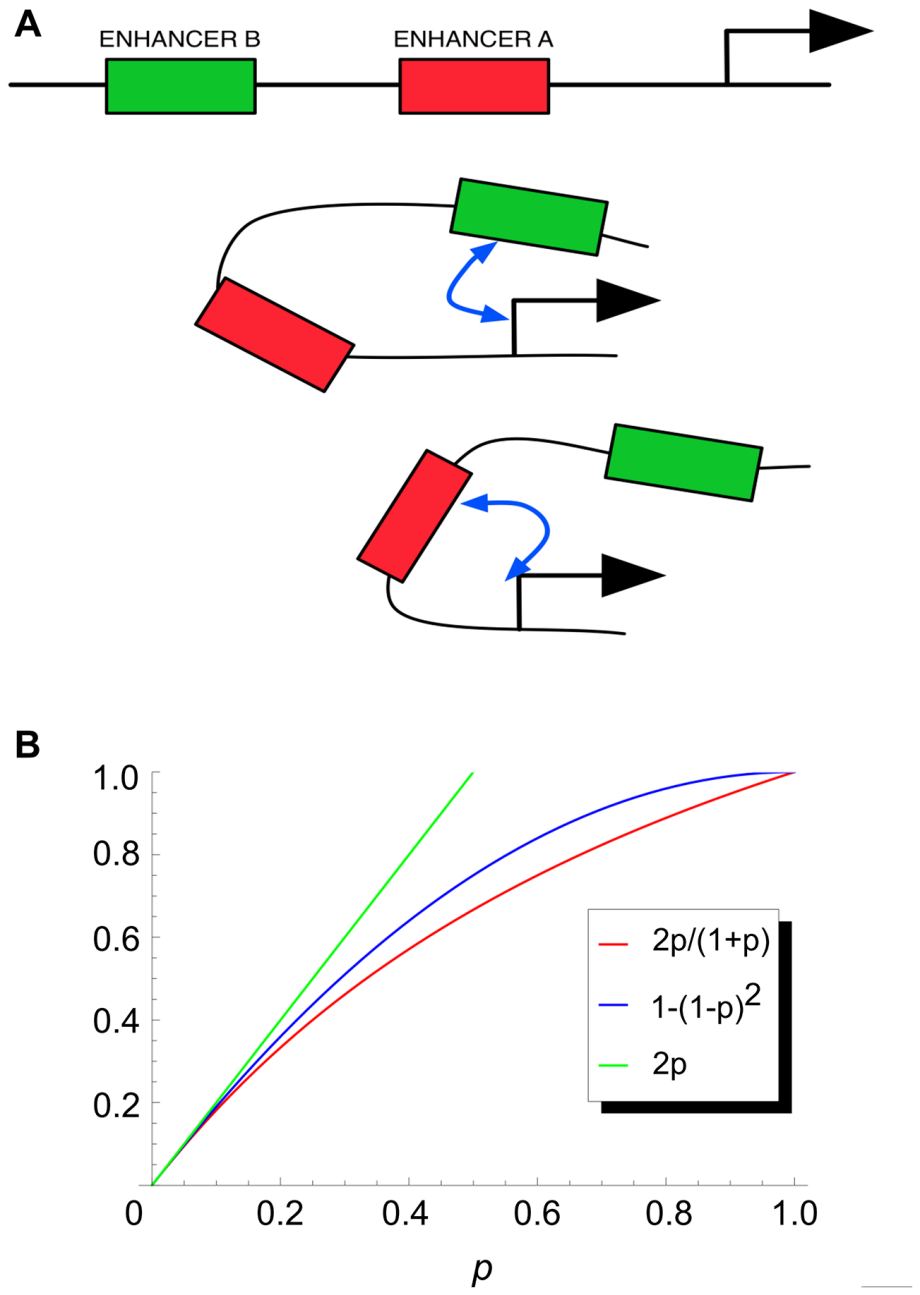
(A) A gene locus with two enhancers (A and B) can be in one of three different configurations of enhancer-promoter interaction: (top) neither enhancer interacts with promoter, (middle) only B interacts and (bottom) only A interacts. In a configuration where A interacts with promoter, B does not interact, and vice versa. (B) Combining contributions from two enhancers. If each enhancer's contribution is given by the gene expression probability *p* due to that enhancer, the combined contribution of the two enhancers (assuming independent interactions with the promoter) is 2p/(1+p), plotted in red. For small values of p, this is well approximated by 2p (green), the sum of their contributions. For larger values of *p*, a better approximation is provided by the function 1−(1−p)^2^, in blue.

One may speculate on biochemical mechanisms that implement the two-tiered readout of the regulatory information at the locus, and the additive aggregator function. An obvious possibility is that each contributing segment interacts with the promoter separately, as shown in Figure 9A. In the example shown, there are two enhancers and three possible configurations of enhancer-promoter interaction. The “Boltzmann weight” of each configuration is assumed to depend only on the enhancer interacting with the promoter in that configuration. Let these weights be 1, η_B_ and η_A_ for the configurations at the top, middle and bottom respectively. Assuming that gene expression ‘
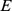
’ is proportional to the total probability ‘
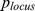
’ of configurations with any enhancer-promoter interaction, we get:
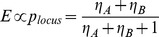
First, let us consider a trans-regulatory context where one of the enhancers (say A) drives expression and the other (say B) does not. This can be formulated as:
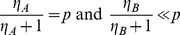
Under these conditions, we get 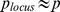, i.e., the contributions of the two enhancers add up to produce the expression driven by the locus. Thus, if a gene is under the control of multiple enhancers and if a single enhancer dominates all others in any particular trans-regulatory context (position along the A/P axis, for pair rule genes), we expect the combined readout of the multiple enhancers to be a sum of their individual readouts.

Now consider a trans-regulatory context where both enhancers A and B (of Figure 9A) have comparable outputs. We may formulate this as:
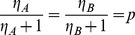
It is easily shown that in this case
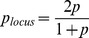
We plot this function, representing the combined readout of the locus, in Figure 9B. For small values of *p* (<0.2), this function is reasonably approximated by 2*p*, indicating that the enhancer contributions add up. Note that a value of *p* = 0.2 does not necessarily mean low gene expression; under the Shea & Ackers theory, expression levels are only *proportional* to *p* as defined here. For larger values of *p*, we see that 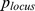 is better approximated by the function 1−(1−p)^2^, which represents the model of enhancer synergy proposed by Perry et al. [43]. In this case, the separate readouts of enhancers do not combine additively. Another scenario in which additivity is not expected is where multiple enhancers can interact simultaneously with the promoter, as is the case in the “long range dominant repression” model of Perry et al. [43]. We explicitly prohibited such a configuration in the model of Figure 9A. We should also note that, the aforementioned assumptions do not preclude overlapping enhancers. Discovery of overlapping enhancers, in this modeling framework, is not therefore a violation of the linearity assumption.

In light of the simplistic arguments presented above, we suggest that the model illustrated in Figure 9A, with the strength of each enhancer-promoter interaction being unaffected by other enhancers in the locus, as a mechanistic basis of the GEMSTAT-GL model. Additivity of enhancer contributions in any given trans-regulatory context can be explained by this model as arising out of (a) one enhancer's contribution dominating all others or (b) each enhancer's contribution being at a relatively low level, i.e., the probability *p* defined above being not close to 1.

### A note on parameter estimation for the locus-level modeling problem

A locus-level model of gene expression requires more precision than an enhancer-level model. The success of an enhancer-level model is typically assessed from its precision in modeling the position of the peaks of the expression domains driven by an enhancer. Consequently, the most successful enhancer-level models produce qualitatively accurate expression patterns for each enhancer but may not capture the peak amplitude of expression domains correctly. That is, relative peak amplitudes of readouts from two enhancers are often inconsistent with model predictions. Another type of imprecision noted in enhancer-level models is the inability to predict the sharp boundaries of expression domains. A locus-level model cannot afford to tolerate such imprecision, especially when it is applied to model complex multi-stripe expression patterns. The two weaknesses of enhancer-level model fits mentioned above can cause our locus-level model to predict qualitatively inaccurate expression patterns (e.g., miss an inter-stripe boundary), and are likely to lead to false regulatory sequence discovery and wrong inference about the roles of TFs. At the same time, the quantitative imprecision in predicted enhancer readouts may be unavoidable at this time due to fundamental limitations of the thermodynamic model, e.g., biochemical mechanisms that are not modeled.

Our strategy of optimizing the thermodynamic parameters for each gene separately was a pragmatic decision made to compensate for the minor inaccuracies of enhancer-level modeling. As shown in Figure S8, when GEMSTAT-GL was optimized without re-training the thermodynamic parameters (thus, the locations and the weights of the windows were the only free parameters in the model), it could still capture the correct locations for five of the seven stripes of *eve* expression but suffered severely in terms of modeling the inter-stripe valleys. Thus, fitting the thermodynamic parameters in a locus-specific manner helps GEMSTAT-GL to achieve the desired accuracy. It is plausible that this strategy might lead to over-fit GEMSTAT-GL for the single intergenic locus being modeled. This is why we performed four different types of negative controls, to demonstrate that the constraints imposed on the parameters during model optimization are strongly guarding us against over-fitting the model for any specific locus.

In a recent study [18], Kim et al. trained thermodynamics-based models on a collection of *eve* enhancers in order to provide deeper insights into combinatorial cis-regulatory logic, which, as they pointed out, is a pre-requisite for locus-level modeling of gene expression. Among other findings, they reported a model that predicts *eve* stripes 2, 3, and 7 from the sequence upstream of the gene, and a different model (i.e., different parameter settings) that predicts stripes 4, 5, and 6 from the sequence downstream of the gene. Their results, in addition to providing insights about functioning of enhancers, highlight the difficulty of modeling the readout of an entire gene locus using pre-determined parameters, even when the models are accurate at the enhancer level. This agrees with our own view mentioned above, and suggests that fitting thermodynamic parameters for individual loci, with appropriate constraints, is a necessary step at the current stage of computational modeling of gene expression from the locus.

## Materials and Methods

### Data collection

We collected enhancers previously reported to regulate the genes *eve*, *h*, *run*, and *gt* from the REDfly database [60]. We also extracted the embryonic expression pattern of each of these genes during the temporal class 5 in nuclear division cycle 14A, from the FlyEx database [57]. The FlyEx database provides the expression level as a function of the A/P axis, obtained after appropriate normalization and averaging over embryos. Expression profiles for known enhancers were derived from respective gene expression profiles based on prior knowledge about which stripes correspond to each enhancer (See Figure S9 for details). We note that, although the real expression profiles being modeled in GEMSTAT and GEMSTAT-GL are shown in the figures as scaled between 0 and 1, it is not a requirement of the models. The output of our models is a probability value, and the gene expression is assumed to be *proportional* to this probability. As such, the models' output (probability) can be scaled and compared against any quantitative expression level. Position weight matrices (PWMs) of all the TFs were taken from the Fly Factor Survey database [78]. Protein concentration profiles of the TFs along the A/P axis were obtained from the FlyEx database [57] and the data set used in [13]. The data sets are made available at http://veda.cs.uiuc.edu/gemstat-gl/index.htm.

### The GEMSTAT model

This is described in detail in our previous work [11], and here we review its main ideas. As per the model, transcriptional regulation takes place through the interaction of three major components: (a) DNA sequence, (b) TF molecules, and (c) the basal transcriptional machinery (BTM). A TF molecule may bind the sequence at any binding site, with site-specific affinity. The BTM may bind at the core promoter of the gene, and it initiates transcription when thus bound. Interactions between bound TF molecules and the BTM (possibly through co-factors, which are not explicitly modeled) determine the occupancy, i.e., probability of binding, of the BTM at the promoter. We assume, following Shea & Ackers [50], that the level of gene expression depends primarily on the rate of transcription initiation, and in particular on the BTM occupancy.

Being a statistical thermodynamics model, GEMSTAT considers an ensemble of molecular configurations of the transcriptional regulatory machinery; each configuration, denoted by *σ*, specifies which sites in the DNA sequence are bound by cognate TFs. For every configuration *σ*, GEMSTAT computes two quantities: (a) the Boltzmann weight of *σ*, denoted by *W(σ)*, and (b) the transcriptional effect of *σ* on the BTM, denoted by *Q(σ)*. The Boltzmann weight *W(σ)* is calculated based on TF concentration and the binding affinity of every bound site in *σ*, which in turn is estimated from the sequence and the TF motif [79]. The transcriptional effect *Q(σ)* represents interactions between bound TF molecules of configuration *σ* and the promoter-bound BTM, and is modeled with free parameters (one per TF) reflecting such interactions. Every configuration *σ* of the regulatory sequence corresponds to two configurations of the entire regulatory apparatus, depending on whether the BTM is bound or not. The relative probability of bound BTM is given by 
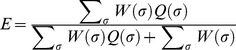
 and the gene expression level is assumed proportional to *E*. GEMSTAT models self-cooperative DNA-binding by any TF through a free parameter (cooperativity term) that is multiplied with *W(σ)* for every occurrence of an adjacent pair of bound sites within a fixed distance from each other.

### Modeling enhancers with GEMSTAT

We performed simultaneous training of GEMSTAT parameters on all known enhancers of a gene. The inputs were the sequence of each enhancer, and the motifs and A/P concentration profiles of nine relevant TFs. GEMSTAT learns optimal parameters such that its predictions for A/P expression pattern of each enhancer matches the corresponding real pattern. GEMSTAT was used in the “DIRECT INTERACTION” mode (see [11]) unless stated otherwise. The number of free parameters is 28 (three per TF, plus one global parameter), and raises concerns about over-fitting. To address this, a “constrained parameter estimation strategy” was used, as described below.

### Constrained parameter estimation strategy

To guard against over-fitting, we used the following model training strategy. We first trained GEMSTAT on ∼40 enhancers with A/P patterned expression [14], while excluding enhancers of the given gene. Training on this large data set greatly constrains the model and rules out over-fitting. We used the parameter values thus obtained as the starting point of the parameter training procedure on regulatory sequences of the given gene. Thereafter, the training procedure was prohibited from altering any parameter's value by more than two fold from its initial value. This strategy ensured that the final model trained on the given gene is largely consistent with a model that reflects other regulatory parts of the genome.

### Modeling a gene locus with GEMSTAT

This was performed just as any individual enhancer would be modeled by GEMSTAT. The inputs were the sequence of the locus, and the motifs and concentration profiles of the nine TFs. GEMSTAT's goal was to learn parameters such that its prediction for the readout of the entire locus matches the gene expression pattern, as quantified by the “weighted Pattern Generating Potential” (w-PGP) score described in the next paragraph. Also, since we claim (see RESULTS) that GEMSTAT is unable to model gene loci, we used an unconstrained parameter estimation strategy where the model training procedure was free to use any parameter values within a reasonable range.

### Evaluation of model predictions using “weighted pattern generating potentials”

Two obvious approaches to assess the agreement between real and predicted expression profiles are the “sum of squared errors” or “correlation coefficient”. However, as shown in our previous work [13], these do not always capture the salient features of an A/P expression pattern. We devised a new scoring function, called “weighted pattern generating potential” (w-PGP) to address this issue. The score is explained in Figure S10 and its legend. It is a modification of the “PGP” score of [13]. The essence of w-PGP is to reward the agreement between real and predicted readouts and penalize the disagreement. We have performed detailed explorations of the w-PGP score in a separate work [52] and found it to be superior to the sum of squared errors or the correlation coefficient.

### GEMSTAT-GL model for predicting gene expression from intergenic sequence

The new quantitative model for predicting gene expression from the entire locus of a gene operates in two tiers (Figure 2). Recall that the inputs to the model are (i) the sequence of the locus, TF motifs, and TF concentration profiles along the A/P axis, and (ii) the gene's expression profile (assumed here to be multiple stripes along the axis). The trained model comprises (i) a set of windows (possibly of varying length, and possibly overlapping each other) in the locus, and their “window weights” (positive numbers), and (ii) values for GEMSTAT parameters reflecting TF-DNA, TF-BTM, and TF-TF interactions. The model's prediction of gene expression is the weighted sum of readouts from every window in the model, the readouts being predicted by GEMSTAT, and the weights being the window weights mentioned above. More specifically, we optimize the following function.
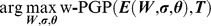
where,

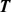
 is a vector representing the expression pattern of the gene being modeled. The 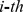 element of this vector denotes the relative level of gene expression at the 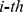 position along the spatial axis;

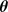
 is a vector representing the thermodynamic parameters;

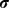
 is a vector representing the window-weights. The 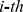 element of this vector denotes the weight of the 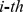 window included in the solution;

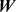
 is a matrix of dimension 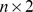, where 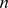 denotes the number of windows included in the solution (hence, 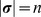). The first and second elements in the 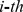 row of 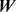
denote the 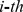 window's location and length, respectively; and

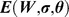
 denotes the dot-product 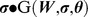, with 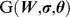 being a matrix whose 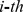 row represents the readout of the 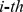 window as predicted by GEMSTAT using the parameters 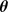. Note that, the location and the length of the 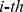 window are specified by the 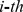 row of 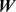.

We describe here the procedure for training the two-tiered model, given its input. The procedure learns optimal values of the GEMSTAT parameters as well as “window weight” parameters (see above) that maximize the w-PGP score between the gene expression profile and the model's prediction. A model is denoted by *M = (* ***W*** *,* ***σ*** *,* ***θ*** *)* where ***W*** is a set of sequence windows from the locus, ***σ*** is the set of window weights, one for each window in ***W***, and ***θ*** is a set of GEMSTAT parameters. The model training happens in two phases. In the beginning, ***θ*** is set to GEMSTAT parameters learned from a large set of known enhancers excluding any known enhancers of the target gene.

#### Phase 1

In the first phase, the algorithm scans the intergenic sequence to find *N = 5* best sequence windows for each stripe in the gene expression pattern. To do so, it examines every window starting at 100 bp intervals in the locus, and of length between 500 bp and 2500 bp. (These are user-configurable parameters.) It scores every window *W* against every stripe *S* of the target gene expression, based on how well the expression read-out of *W* (predicted by GEMSTAT) fits the expression profile of *S*. The fit is quantified by the w-PGP score. At the end of this phase, the algorithm has found a set of *N* best windows for each stripe *S*, denoted by *C(S)*.

#### Phase 2

Next, the algorithm iteratively selects windows to include in the model, and learns their corresponding window weights. In the *i^th^* iteration, it builds a model *M_i_ = (* ***W_i_*** *,* ***σ_i_*** *,* ***θ*** *)* for the first *i* stripes of gene expression. A pseudo-code is provided next.

Given: GEMSTAT parameters ***θ*** and a candidate set of windows *C(S)* for every stripe *S*.

Initialization: ***W_0_***: = NULL; *BESTSCORE_0_*: = 0.

For *i*: = 1 to *K* (the number of stripes in gene expression pattern) do:

1. ***W_i_***: = ***W_i−1_***; *BESTSCORE_i_*: = *BESTSCORE_i−1_*;
2. For each window *w* in *C(S_i_)* do
  - Define a new set *W′* = ***W_i_*** U {*w*}
  - Let ***σ*** be a set of window weights, one weight for each window in W′. Let Score_i_(***W′***, ***σ***) denote the w-PGP score that compares (i) the two-tiered model predictions using windows of ***W′*** and window weights ***σ***, and (ii) the gene expression pattern limited to the first *i* stripes or expression domains. (The stripes were considered arbitrarily from anterior to posterior.)
  - Find ***σ*** that maximizes *Score_i_(* ***W′*** *,* ***σ*** *)* over all possible ***σ***. This maximization is performed through alternating between the Simplex and the Gradient Descent algorithms for numerical optimization. Denote max***_σ_*** *Score_i_(* ***W′*** *,* ***σ*** *)* by *Score(w)*.
3. Let w^*^ denote the window that maximizes *Score(w)* in the previous step.
4. If Score(w^*^) is greater than *BESTSCORE_i_*, then
  - ***W_i_***: = ***W_i_*** U {w^*^}
  - *C(S_i_)*: = C(S_i_)\w*
  - *BESTSCORE_i_*: = Score(w^*^)
  - Loop back to (2).

At the end of this phase, a model *M = (* ***W*** *,* ***σ*** *,* ***θ*** *)* has been found for the entire expression pattern. Now, the GEMSTAT parameters ***θ*** are retrained while keeping ***W*** and ***σ*** fixed. The algorithm then loops back to Phase 1. It iterates through these two phases until a constant number *N_I_* of iterations have been completed or the improvement in the model's w-PGP score is less than a small constant *δ>0*. We set *N_I_ = 100* and *δ = 10^−4^* for training the models in this paper.

We note that, the GEMSTAT model [11] is used in two contexts while training GEMSTAT-GL. First, to compute the initial estimates for GEMSTAT-GL's thermodynamic parameters, we optimize GEMSTAT for ∼40 A/P patterning enhancers associated with genes other than the gene being modeled. The objective function used to optimize GEMSTAT for this purpose was the average of the w-PGP scores of all enhancers in the dataset. Starting from these initial values, GEMSTAT-GL then searches the parameter space for better estimates of its thermodynamic parameters. In the second scenario, GEMSTAT is used to compute the readout of every window that GEMSTAT-GL examines within the locus (i.e., in Phase 1 described above). In this case, GEMSTAT-GL inputs its thermodynamic parameters and a sequence window to GEMSTAT, which then outputs the readout of the given window resulting from the input parameters.

### Control experiments

(1) One of the negative control experiments involved modeling a gene's expression pattern from a randomly generated sequence of the same length as the gene locus. The random sequence was generated by independently sampling each nucleotide from a common frequency distribution. (2) Another negative control experiment involved modeling a “random” expression pattern from the sequence of a gene locus. Random expression patterns were generated based on the gene's real expression pattern, as follows. First, for any axial position, let us define the gene to be OFF if the expression value is less than 0.5 and ON otherwise. Then, for a gene *G* whose actual expression profile has *N* stripes and *K* axial positions where it is ON, we defined a “random” expression profile as one where: (a) the number of stripes is a randomly chosen number between *N/2* and *N*, (b) the stripes are located randomly along the A/P axis, and (c) there are *K* data points where it is ON. Computation of such a random expression is detailed in Figure S11. (3) In the final set of negative control experiments we used a “variant” of a gene's locus, where the TF binding sites were relocated to randomly selected positions within the locus, to model the gene's expression pattern.

### Constructing a regulatory network of TF-stripe interaction through *in silico* TF knockdown

In order to infer edges in the TF-stripe interaction networks, we repeated the following steps for each TF. First the relative concentration of the TF being examined was set to zero at every position along the A/P axis (the relative concentration profiles of all other TFs were left unchanged). This was essentially an *in silico* knock-down of the TF being examined. A GEMSTAT-GL model trained earlier using real data for the target gene was then re-run on this new data (but not optimized anew). We then observed the resulting output and inferred edges in the network as follows. If knock-down of the TF was found to weaken a stripe's expression (by at least 2%), we inferred an activation edge from the TF to the stripe. In case the knock-down was found to strengthen the stripe's expression (by at least 2%) and/or shift the stripe's boundary (by at least 1% of the A/P axis at the position of half-maximum peak expression), we inferred a repression edge.

### Sampling the two-tiered model

As noted above, a model is denoted by *M = (* ***W*** *,* ***σ*** *,* ***θ*** *)* where ***W*** is a set of sequence windows from the locus, ***σ*** is the set of window weights, one for each window in ***W***, and ***θ*** is a set of GEMSTAT parameters. We described above a local search algorithm to find the optimal model. We also performed MCMC sampling of the space of all possible windows and window-weights, i.e., *(* ***W*** *,* ***σ*** *)* for a global examination of the expression contributions of segments in the locus.

#### Sample space

Each sample is an *extended* weight vector **σ** that has one real number for every possible window in the locus. Recall that this includes windows of length between 500 and 2500 (in increments of 50), with start positions that are multiples of 100 bp. Note also that any **σ** corresponds to a particular model *M*_σ_: the window set ***W*** is determined by the non-zero weights in **σ**, and the GEMSTAT parameters ***θ*** are assumed fixed. The w-PGP score of model *M*_σ_ is denoted by Score(**σ**), and the MCMC attempts to sample **σ** with probability proportional to Score(**σ**).

#### Sampling algorithm

We used the Metropolis-Hastings algorithm to sample **σ**. The allowed moves from a current sample ***σ_i_*** are determined as follows. Let ***b_i_*** be a bit vector of the same dimensionality as ***σ_i_*** and its *j^th^* bit being defined as *b_ij_* = 1 if *σ_ij_*>0 and *b_ij_* = 0 otherwise. That is, ***b_i_*** indicates which windows have positive weights in ***σ_i_***. The samples reachable in one move from the current sample ***σ_i_*** (with bit vector ***b_i_***) are those with bit vectors within a Hamming distance of 2 from ***b_i_***. In other words, any move adds or deletes at most two windows from consideration in the first tier of the model. The proposal distribution of the Metropolis Hastings algorithm is described next. Given a current sample ***σ_i_*** (with bit vector ***b_i_***), we choose two bits at random and toggle each bit with probability 1/2. This samples a bit vector ***b_j_*** that is (a) identical to ***b_i_*** with probability ¼, (b) 1 Hamming distance from ***b_i_*** with probability ½, and (c) 2 Hamming distance from ***b_i_*** with probability ¼. All bit vectors with a particular Hamming distance are equally likely. There are *L* = |*b_i_*| of these at Hamming distance 1, and 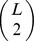 of these at Hamming distance 2. The newly sampled bit vector ***b_j_*** is then used as the “shape vector” of a Dirichlet distribution, from which a probability vector is sampled. This is the newly sampled weight vector ***σ_j_***. As prescribed by the Metropolis Hastings algorithm, this proposed sample ***σ_j_*** is then accepted with probability min(1, Score(*σ_j_*)/Score(*σ_i_*)).

### Constructing heatmaps to study enhancer interactions

Our goal was to probe potential interactions between binding sites from two different enhancers. In particular, we wanted to determine if interpreting the sites of one enhancer together with sites from another enhancer leads to better model predictions than the baseline of GEMSTAT-GL where each enhancer is interpreted independently. A natural way to represent such potential interactions is with a *hypergraph*. Hypergraphs generalize the concept of graphs by allowing each edge (called “hyperedge”) to represent a relationship shared among more than two nodes. In our formulation, every binding site in every enhancer is a node in a hypergraph, and any subset of sites from two different enhancers defines a hyperedge. The evidence in favor of that subset of sites being interpreted together, as if they were sites in the same enhancer, is the weight of the hyperedge. Such weights can be negative also, indicating that the particular subset of sites if interpreted together will make model predictions worse. We limited our attention to hyperedges defined by including (a) all sites of one enhancer and (b) sites within a sub-segment of a different enhancer, thus simplifying the space of enhancer interactions considered. Our model-based predictions of potential interactions (or avoidance of interactions) can be captured by this weighted hypergraph. However, a hypergraph is hard to visualize and less likely to lead to biological insights via direct examination. We therefore mapped the constructed hypergraph to a weighted graph where the weight of every edge represents the effect of allowing interaction between the two binding sites that the edge represents. Visualization of the edge weights of this graph through heatmaps then revealed how any binding site could affect the readout of any enhancer in our model.

#### Hypergraph construction

For a gene *g*, suppose the GEMSTAT-GL model selects *n* contributing enhancers *C_1_, C_2_, …, C_n_*. Let *SITES(C_i_)* denote the set of TF binding sites in enhancer *C_i_*. Then, for every binding site in every set *SITES(C_i_)*, we include one node in a hypergraph. There are two types of hyperedges in our hypergraph. First, every subset of *SITES(C_i_)* constitutes one hyperedge, and every such hyperedge was assigned a weight of zero. Each of the remaining hyperedges represents a collection of binding sites from two different enhancers, and was constructed as follows. Let *e_h_* denote a hyperedge that consists of binding sites from enhancers *C_1_* and *C_2_*. Then the hyperedge *e_h_* would include all the binding sites of one enhancer (say, *C_1_*) and between one and five contiguous binding sites of the other enhancer (*C_2_* in this case). For each hyperedge *e_h_* constructed in this way, we optimized a new GEMSTAT-GL model where the contributing enhancers are *C_2_*, … *C_n_*, as well as the newly constructed set of sites *e_h_* treated as an “enhancer”. The difference between the w-PGP score of this new model and the original model learned for gene *g* was then assigned as the weight of *e_h_*.

#### Mapping hypergraph to graph

A graph was constructed with the same nodes as that in the hypergraph, with an edge for each pair of nodes. The weight of an edge was computed by averaging the weight of every hyperedge where the corresponding pair of nodes appeared. This approach of approximating a hypergraph through a graph was discussed in detail in [80]. By construction, this graph has the property that the edge between node *i* and node *j* has the same weight for all nodes *j* corresponding to sites in the same enhancer.

## Supporting Information

Figure S1 Results of failed attempts to model the seven-striped expressions of *eve* (left panel), *h* (middle panel), and *run* (right panel) from their respective intergenic regions. (A,B): GEMSTAT-predicted readout of the entire locus in the ‘Direct Interaction’ (A) and the ‘Short Range Repression’ (B) modes respectively. (C) GEMSTAT-predicted readout of the concatenation of all known enhancers of the gene. No enhancer has been reported to date for stripes 2, 4, and 6 of *run*. We therefore tried modeling only stripes 1, 3, 5, and 7 from the concatenation of the known enhancers of *run*.
(TIF)

Figure S2 Role of the weight parameters in the two-tiered model. For each gene (column), the top panel shows the un-scaled readouts of individual segments selected by the model, the middle panel shows an un-weighted summation of these readouts (green, compared to real expression profile in red), and the bottom panel shows the weighted summation reported by our model along with the weight of each GEMSTAT-GL selected window in the inset.
(TIF)

Figure S3 Extent of overlap between REDFly enhancers and GEMSTAT-GL selected windows. GEMSTAT-GL selected 50 windows to model the 27 genes mentioned in Table S1, Figure 4, and Figure 5. Out of these 50 windows, 39 were found to overlap with REDFly enhancers. No window overlapped with two REDFly enhancers and no two windows overlapped with the same REDFly enhancer. (A) Each bar represents the length of a REDFly enhancer (normalized to 100%) mentioned in the vertical axis. The green (red, resp.) bar shows the percentage of basepairs in the REDFly enhancer that were found to be included (not included, resp.) in the overlapping GEMSTAT-GL window. (B) Each bar represents the length of a GEMSTAT-GL window (normalized to 100%) that overlaps with the REDFly enhancer mentioned in the vertical axis. The green (red, resp.) bar shows the percentage of basepairs in the window that are common (not common, resp.) with the overlapping REDFly enhancer. For example, for the enhancer tll_P2, panel (A) shows that GEMSTAT-GL used such a window, say W, in modeling the *tll* gene that completely contains the tll_P2 enhancer, while panel (B) shows that the tll_P2 sequence comprises about 90% of W.
(TIF)

Figure S4 Results of “negative control” experiments. (A) Modeling a gene's expression from the intergenic region of a different gene. In each case, the model was trained to fit the real expression profile of a gene (red) using sequence from a different gene's locus. The best-fit predictions (green) did not match the real profiles well. (B) Modeling random expression patterns (red) from the intergenic sequences of *eve*, *h*, and *run*. Best-fit predictions are shown in green. (C) Modeling real expression patterns (red) from random sequences of the same length as the locus of the corresponding gene. Best-fit predictions are shown in green.
(TIF)

Figure S5 Histograms reflecting the empirical distributions of w-PGP scores computed from a negative control experiment (repeated 100 times) where a gene's expression pattern was modeled from its own locus but the binding sites within the locus were randomly relocated. (A)–(D) Histograms for *eve*, *h*, *run*, and *gt*, respectively. Each histogram drawn with red bars was obtained from models trained in the negative control experiment, while the green bar corresponds to the original model.
(TIF)

Figure S6 Results of MCMC sampling for the genes *h* (A) and *run* (B) along with the REDFly enhancers and GEMSTAT-GL selected windows viewed in Genome Browser. Semantics of the plots are described in the legend of Figure 6.
(TIF)

Figure S7 Heatmap visualizations of the changes in GEMSTAT-GL's goodness-of-fit owing to interactions between the enhancers selected for the genes (A) *h*, (B) *run*, and (C) *gt*. Semantics of the heatmaps are explained in the legend of Figure 8.
(TIF)

Figure S8 (A) Seven-stripe expression pattern of *eve* (red) and GEMSTAT-GL prediction (green) when thermodynamic parameters were kept fixed during model fitting. (B–F) Model-predicted readouts (green) of individual windows automatically discovered by GEMSTAT-GL. These readouts are aggregated by the model using weighted summation, to produce the locus-level readout shown in (A).
(TIF)

Figure S9 Steps in extracting enhancer expression profile from experimentally characterized gene expression profile.
(TIF)

Figure S10 An overview of the ‘weighted pattern generating potential’ (w-PGP) scheme to score model predictions (design choices have been explained in [82]). The red and the green curves depict 16 data points of a real expression pattern and the corresponding predicted expression pattern, respectively. While scoring the predicted expression for similarity to the real expression, w-PGP determines a reward and a penalty for each data point of the predicted expression. Reward is based on expression that has been predicted correctly and penalty is based on expression that has been predicted erroneously (i.e., over expression or missed expression). For example, as the reward term at data point 10 (where the model missed some portion of the real expression), w-PGP uses the product of c_10_ (predicted expression) and r_10_ (real expression). As the penalty term at the same data point, w-PGP uses the product of e_10_ (missed expression) and p_10_ (the maximum possible value of missed expression). On the other hand, for data point 7 (where there is an over expression), w-PGP does not assign any penalty but computes the reward term as the product of c_7_ (predicted expression) and r_7_ ( = c_7_).
(TIF)

Figure S11 Steps in computing a random expression pattern corresponding to the real expression pattern of a gene G, for setting up one kind of “negative control” experiments. If G has N stripes and is expressed in K axial positions, we first select a random number (denoted by ‘stripe count’) between N/2 and N as the number of stripes of the random expression. Next, we compute stripe count number of segments such that the lengths of those segments sum up to K. These segments denote the widths of the stripes in our random expression. Similarly, assuming that G is not expressed in K^0^ number of axial positions, we compute stripe count +1 number of segments such that the lengths of those segments sum up to K^0^. These segments denote the widths of the gaps between successive stripes in our random expression. We then start with a gap, and concatenate the stripes and the gaps alternately to generate the desired random expression. In a final step, we smooth this random expression through a logistic function.
(TIF)

Table S1 Intergenic locus sizes of the 27 genes modeled in the study.
(PDF)

Table S2 w-PGP scores of GEMSTAT-GL predicted expression patterns.
(PDF)

Text S1 Lack of estimates for pair-rule TF parameters constrains the initial focus on primary pair-rule genes.
(DOCX)
