# Supplementary figures and images for: Quantitative Modeling of a Gene's Expression from Its Intergenic Sequence
Source: PLoS Comput Biol. 2014 Mar 6;10(3):e1003467. doi: 10.1371/journal.pcbi.1003467 (PMC3945089; doi:10.1371/journal.pcbi.1003467)

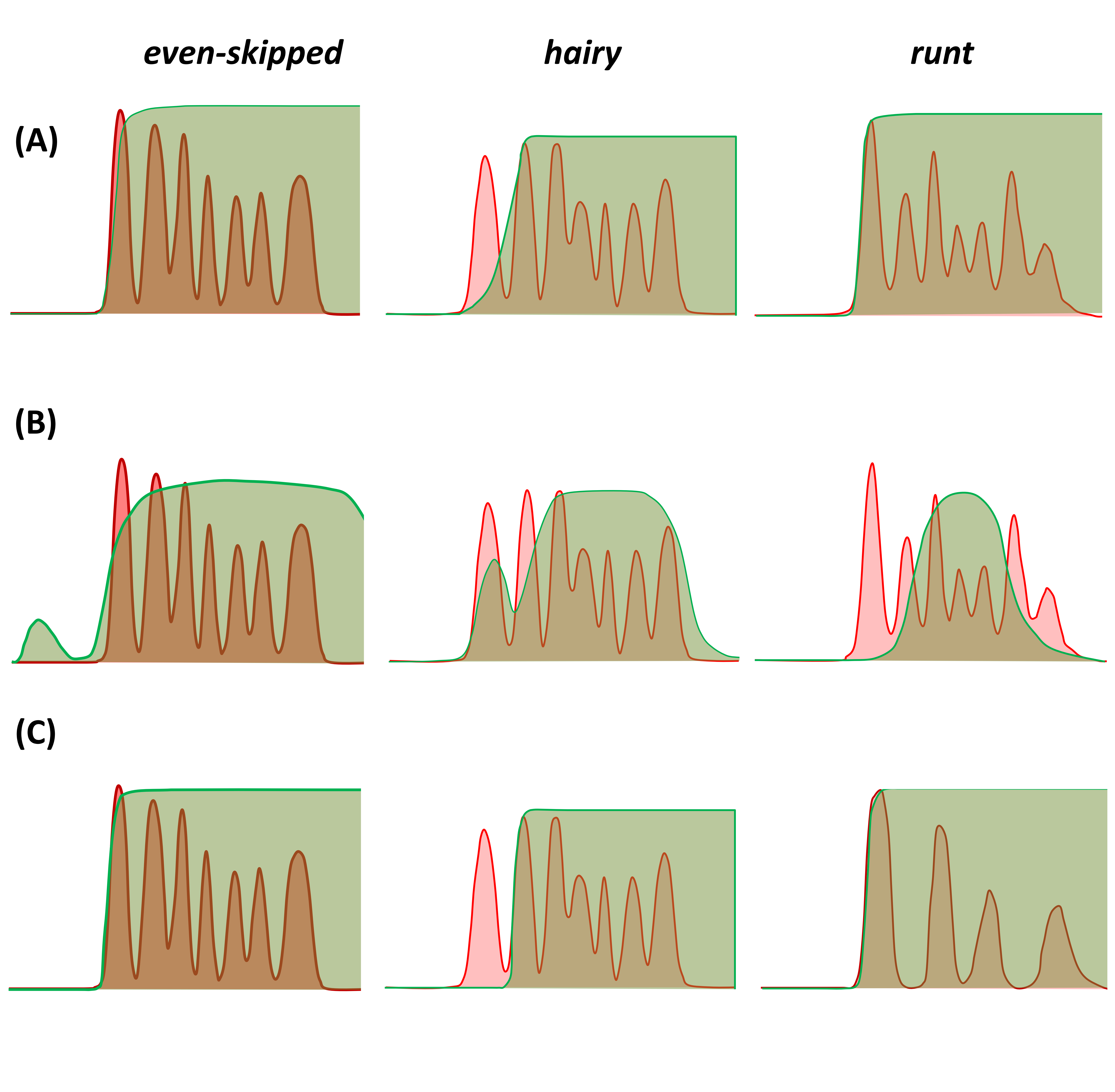

Supplement: Figure S1 — Results of failed attempts to model the seven-striped expressions of eve (left panel), h (middle panel), and run (right panel) from their respective intergenic regions. (A,B): GEMSTAT-predicted readout of the entire locus in the ‘Direct Interaction’ (A) and the ‘Short Range Repression’ (B) modes respectively. (C) GEMSTAT-predicted readout of the concatenation of all known enhancers of the gene. No enhancer has been reported to date for stripes 2, 4, and 6 of run. We therefore tried modeling only stripes 1, 3, 5, and 7 from the concatenation of the known enhancers of run. (TIF) [file pcbi.1003467.s001.tif]

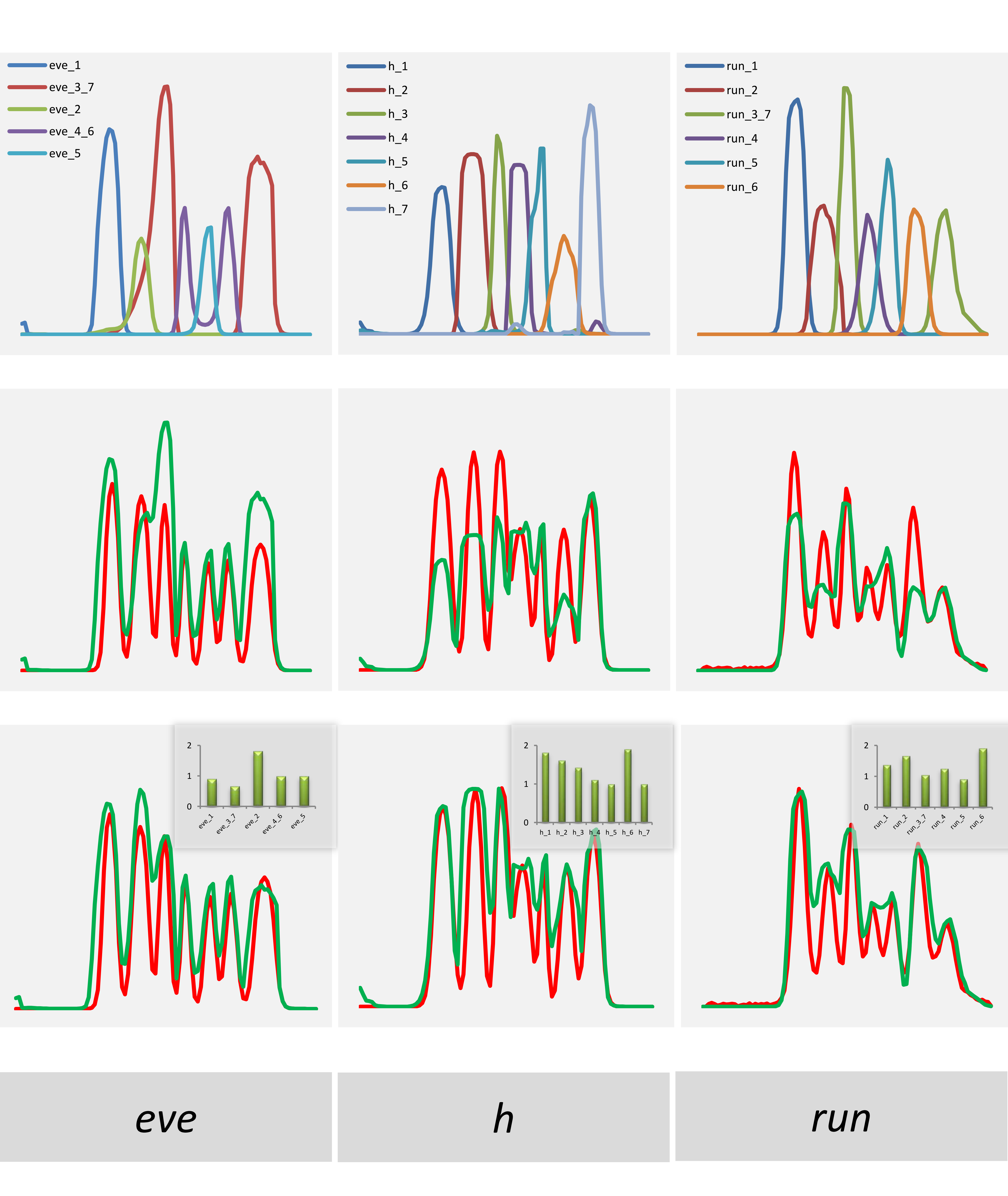

Supplement: Figure S2 — Role of the weight parameters in the two-tiered model. For each gene (column), the top panel shows the un-scaled readouts of individual segments selected by the model, the middle panel shows an un-weighted summation of these readouts (green, compared to real expression profile in red), and the bottom panel shows the weighted summation reported by our model along with the weight of each GEMSTAT-GL selected window in the inset. (TIF) [file pcbi.1003467.s002.tif]

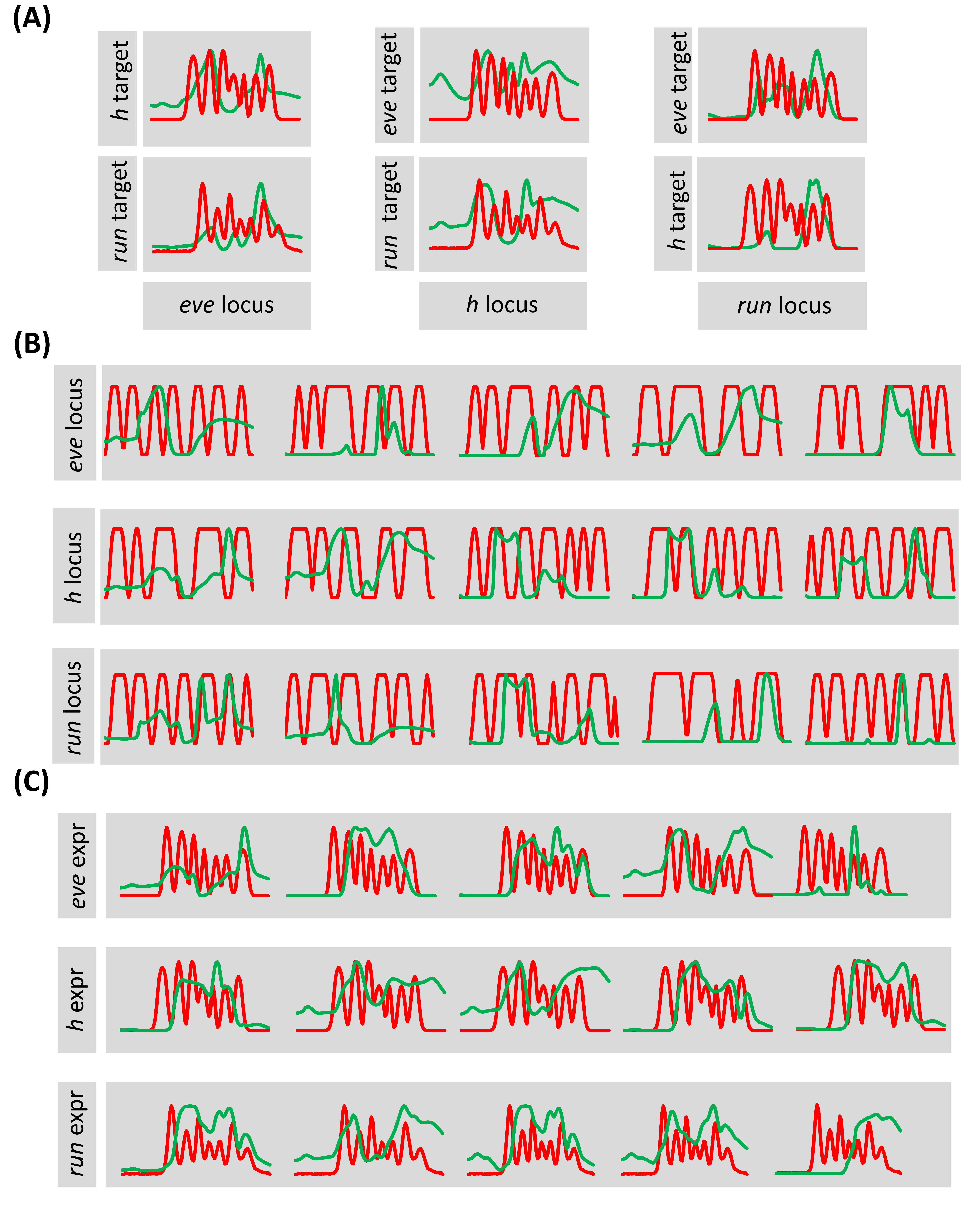

Supplement: Figure S4 — Results of “negative control” experiments. (A) Modeling a gene's expression from the intergenic region of a different gene. In each case, the model was trained to fit the real expression profile of a gene (red) using sequence from a different gene's locus. The best-fit predictions (green) did not match the real profiles well. (B) Modeling random expression patterns (red) from the intergenic sequences of eve, h, and run. Best-fit predictions are shown in green. (C) Modeling real expression patterns (red) from random sequences of the same length as the locus of the corresponding gene. Best-fit predictions are shown in green. (TIF) [file pcbi.1003467.s004.tif]

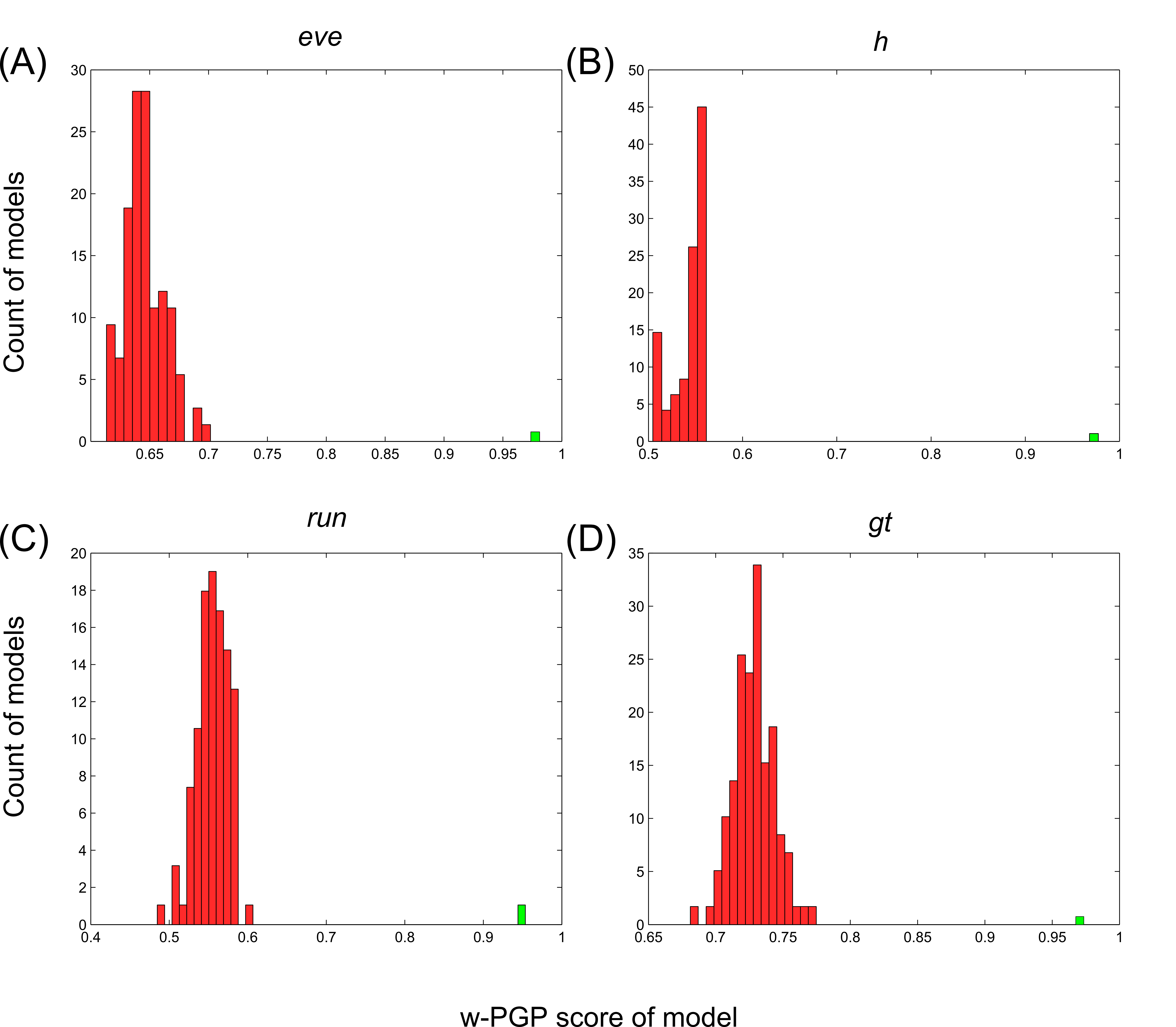

Supplement: Figure S5 — Histograms reflecting the empirical distributions of w-PGP scores computed from a negative control experiment (repeated 100 times) where a gene's expression pattern was modeled from its own locus but the binding sites within the locus were randomly relocated. (A)–(D) Histograms for eve, h, run, and gt, respectively. Each histogram drawn with red bars was obtained from models trained in the negative control experiment, while the green bar corresponds to the original model. (TIF) [file pcbi.1003467.s005.tif]

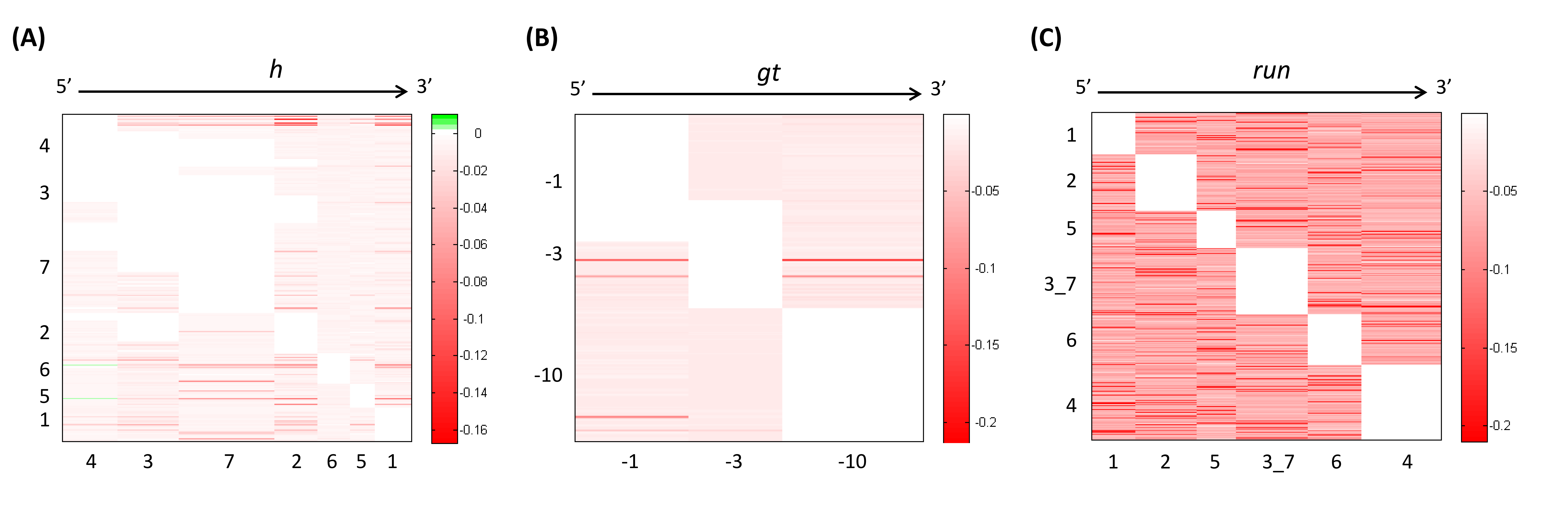

Supplement: Figure S7 — Heatmap visualizations of the changes in GEMSTAT-GL's goodness-of-fit owing to interactions between the enhancers selected for the genes (A) h, (B) run, and (C) gt. Semantics of the heatmaps are explained in the legend of Figure 8. (TIF) [file pcbi.1003467.s007.tif]

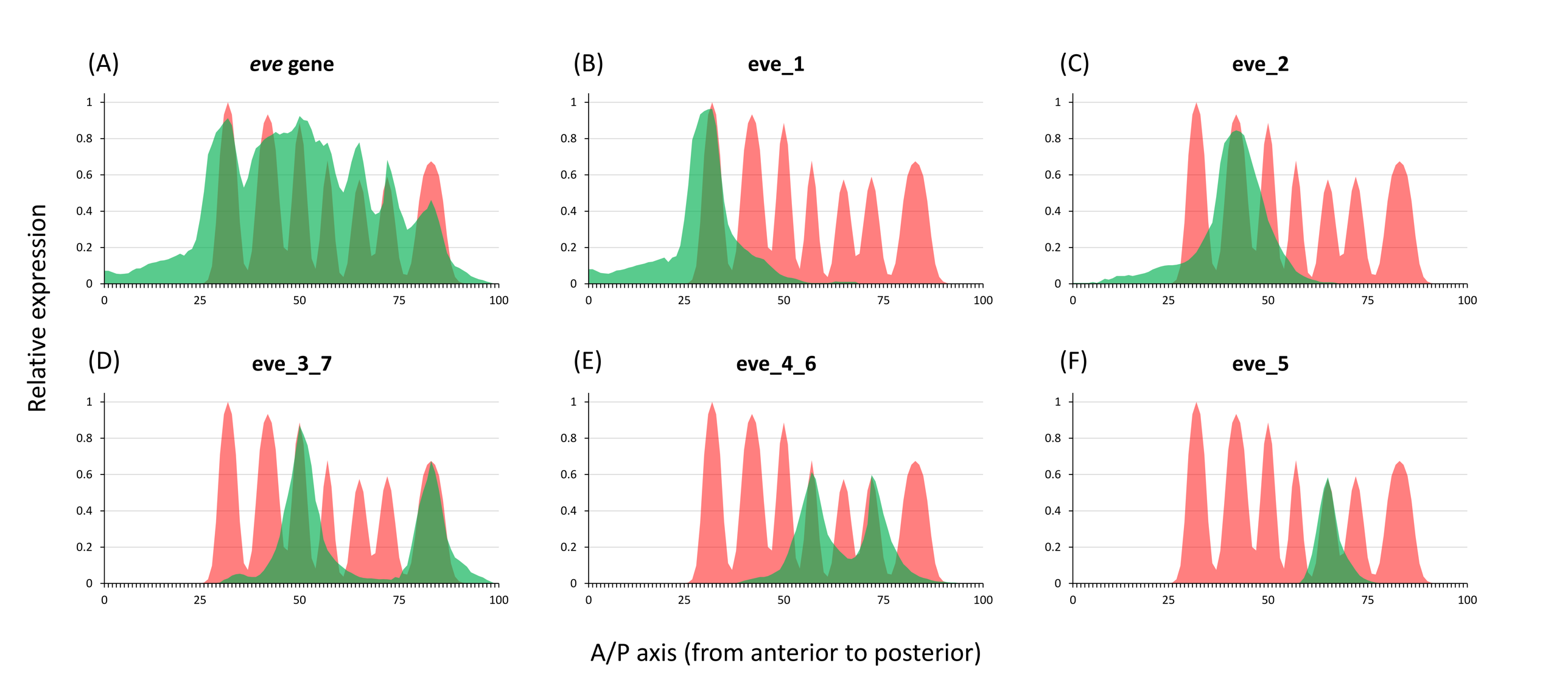

Supplement: Figure S8 — (A) Seven-stripe expression pattern of eve (red) and GEMSTAT-GL prediction (green) when thermodynamic parameters were kept fixed during model fitting. (B–F) Model-predicted readouts (green) of individual windows automatically discovered by GEMSTAT-GL. These readouts are aggregated by the model using weighted summation, to produce the locus-level readout shown in (A). (TIF) [file pcbi.1003467.s008.tif]

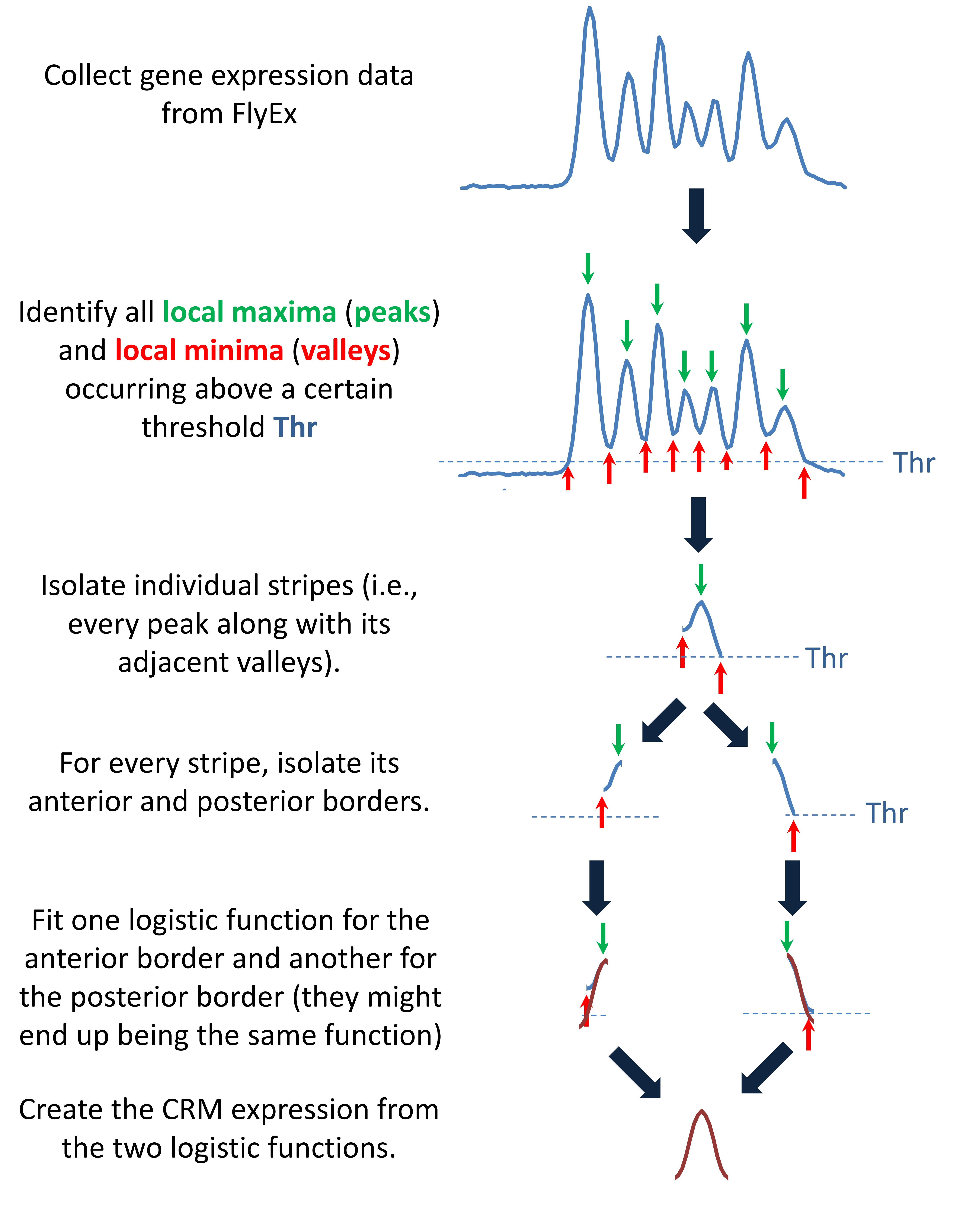

Supplement: Figure S9 — Steps in extracting enhancer expression profile from experimentally characterized gene expression profile. (TIF) [file pcbi.1003467.s009.tif]

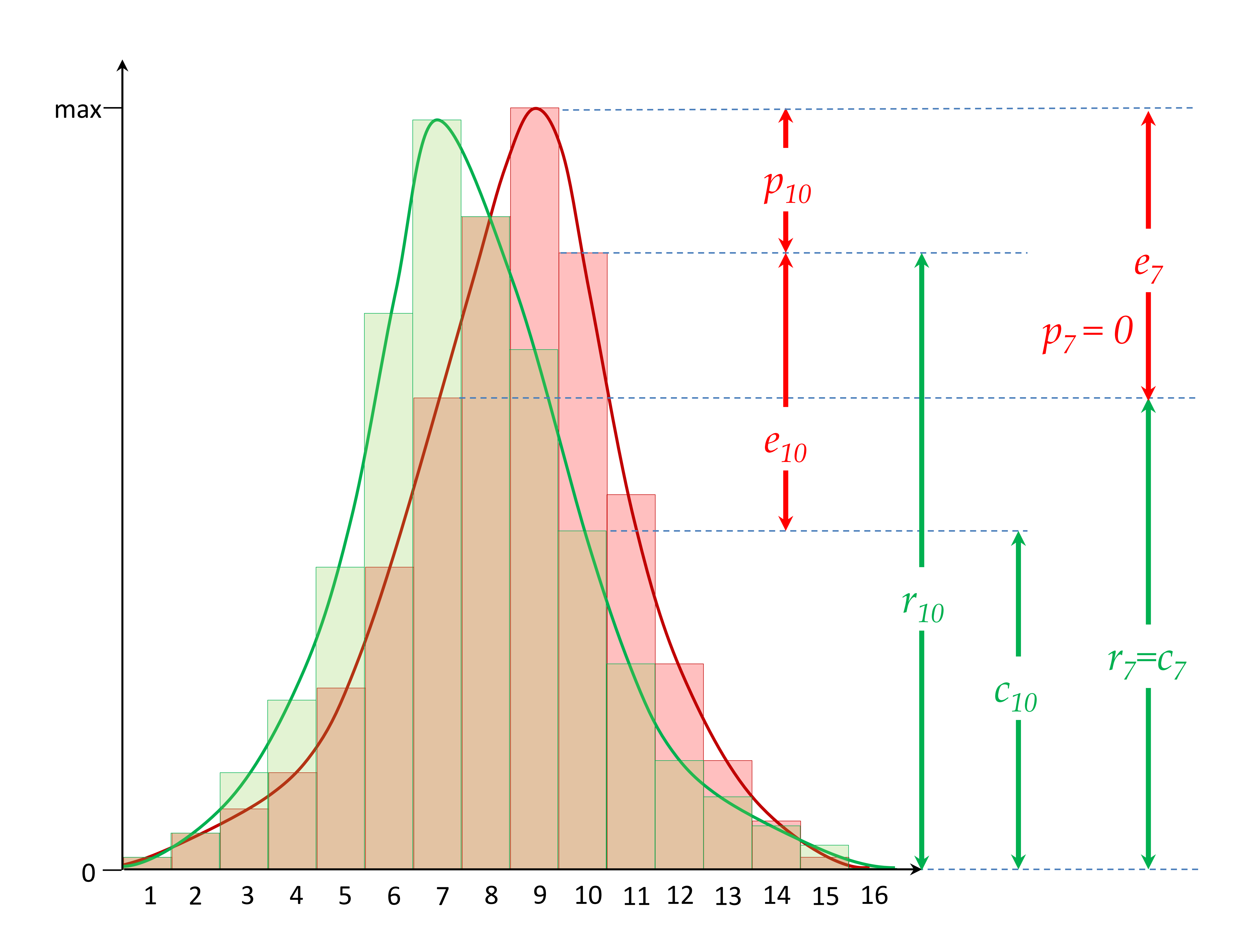

Supplement: Figure S10 — An overview of the ‘weighted pattern generating potential’ (w-PGP) scheme to score model predictions (design choices have been explained in [82]). The red and the green curves depict 16 data points of a real expression pattern and the corresponding predicted expression pattern, respectively. While scoring the predicted expression for similarity to the real expression, w-PGP determines a reward and a penalty for each data point of the predicted expression. Reward is based on expression that has been predicted correctly and penalty is based on expression that has been predicted erroneously (i.e., over expression or missed expression). For example, as the reward term at data point 10 (where the model missed some portion of the real expression), w-PGP uses the product of c10 (predicted expression) and r10 (real expression). As the penalty term at the same data point, w-PGP uses the product of e10 (missed expression) and p10 (the maximum possible value of missed expression). On the other hand, for data point 7 (where there is an over expression), w-PGP does not assign any penalty but computes the reward term as the product of c7 (predicted expression) and r7 ( = c7). (TIF) [file pcbi.1003467.s010.tif]
