# Supplementary material for: Quantitative Modeling of a Gene's Expression from Its Intergenic Sequence
Source: PLoS Comput Biol. 2014 Mar 6;10(3):e1003467. doi: 10.1371/journal.pcbi.1003467 (PMC3945089; doi:10.1371/journal.pcbi.1003467)
Supplement: Table S1 — Intergenic locus sizes of the 27 genes modeled in the study. (PDF) [file pcbi.1003467.s012.pdf]

Table S1

Intergenic locus sizes of the 27 genes modeled in the study

Gene	Intergenic locus size (in kilobases)
eve	16.7
h	70
run	58
gt	16.9
bcd	9.8
btd	44
cad	20.7
cnc	37
D	56
ems	68
fkh	54
ftz	46
hb	21
hkb	20.2
kni	69
knrl	97.5
Kr	58
nub	111.8
oc	71
odd	39
opa	45
pdm2	63
prd	16.3
slp1	23.5
slp2	36
tll	25.8
ttk	26.4
