# Supplementary material for: Quantitative Modeling of a Gene's Expression from Its Intergenic Sequence
Source: PLoS Comput Biol. 2014 Mar 6;10(3):e1003467. doi: 10.1371/journal.pcbi.1003467 (PMC3945089; doi:10.1371/journal.pcbi.1003467)
Supplement: Table S2 — w-PGP scores of GEMSTAT-GL predicted expression patterns. (PDF) [file pcbi.1003467.s013.pdf]

Table S2

w-PGP scores of GEMSTAT-GL predicted expression patterns

Gene	w-PGP score (min: 0, max: 1)
eve	0.98
h	0.97
run	0.96
gt	0.97
bcd	0.97
btd	0.97
cad	0.75
cnc	0.91
D	0.94
ems	0.97
fkh	0.94
ftz	0.68
hb	0.79
hkb	0.97
kni	0.88
knrl	0.92
Kr	0.78
nub	0.91
oc	0.98
odd	0.60
opa	0.84
pdm2	0.97
prd	0.78
slp1	0.95
slp2	0.90
tll	0.95
ttk	0.48
