# Supplementary material for: Quantitative Modeling of a Gene's Expression from Its Intergenic Sequence
Source: PLoS Comput Biol. 2014 Mar 6;10(3):e1003467. doi: 10.1371/journal.pcbi.1003467 (PMC3945089; doi:10.1371/journal.pcbi.1003467)
Supplement: Text S1 — Lack of estimates for pair-rule TF parameters constrains the initial focus on primary pair-rule genes. (DOCX) [file pcbi.1003467.s014.docx]

**TEXT S1**

**Lack of estimates for pair-rule TF parameters constrains the initial focus on primary pair-rule genes**

We note that, several genes omitted in our initial study, e.g., *fushi-tarazu* (*ftz*), *odd-skipped* (*odd*), and *paired* (*prd*) are expressed in the same complex patterns (termed as “pair-rule” patterns) as those of our chosen genes. During early embryogenesis, *eve*, *h*, and *run* are patterned without the input from any other pair-rule gene (hence they are referred to as “primary” pair-rule genes) while *ftz*, *odd*, and *prd* depend on other pair-rule genes for their expression (the reason why they are referred to as “secondary” pair-rule genes) [[1-3](#_ENREF_1)]. We omitted the secondary pair-rule genes in our initial study because including the pair-rule TFs among our inputs would presumably lead to models that underestimate the role of the gap inputs and unreasonably leverage the pair-rule TFs’ spatial patterns to model the expression pattern of a given pair-rule gene. This could be circumvented if reasonable estimates for the pair-rule TFs’ parameter values were known; our “constrained” parameter estimation strategy (see Methods) would then adhere to those estimates. However, estimating the parameters for the pair-rule TFs is an unexplored problem to date; all the state of the art sequence to expression models focus on early enhancers which integrate inputs mainly from the maternal and gap TFs.

**REFERENCES**

1. Carroll SB: **Zebra patterns in fly embryos: activation of stripes or repression of interstripes?** *Cell* 1990, **60:**9-16.

2. Sanchez L, Thieffry D: **Segmenting the fly embryo: a logical analysis of the pair-rule cross-regulatory module.** *J Theor Biol* 2003, **224:**517-537.

3. Prazak L, Fujioka M, Gergen JP: **Non-additive interactions involving two distinct elements mediate sloppy-paired regulation by pair-rule transcription factors.** *Dev Biol* 2010, **344:**1048-1059.
